# A Novel Family of *Toxoplasma* IMC Proteins Displays a Hierarchical Organization and Functions in Coordinating Parasite Division

**DOI:** 10.1371/journal.ppat.1001094

**Published:** 2010-09-09

**Authors:** Josh R. Beck, Imilce A. Rodriguez-Fernandez, Jessica Cruz de Leon, My-Hang Huynh, Vern B. Carruthers, Naomi S. Morrissette, Peter J. Bradley

**Affiliations:** 1 Department of Microbiology, Immunology and Molecular Genetics, University of California, Los Angeles, Los Angeles, California, United States of America; 2 Department of Molecular Biology and Biochemistry, University of California, Irvine, Irvine, California, United States of America; 3 Department of Microbiology and Immunology, University of Michigan School of Medicine, Ann Arbor, Michigan, United States of America; University of Geneva, Switzerland

## Abstract

Apicomplexans employ a peripheral membrane system called the inner membrane complex (IMC) for critical processes such as host cell invasion and daughter cell formation. We have identified a family of proteins that define novel sub-compartments of the *Toxoplasma gondii* IMC. These IMC Sub-compartment Proteins, ISP1, 2 and 3, are conserved throughout the Apicomplexa, but do not appear to be present outside the phylum. ISP1 localizes to the apical cap portion of the IMC, while ISP2 localizes to a central IMC region and ISP3 localizes to a central plus basal region of the complex. Targeting of all three ISPs is dependent upon N-terminal residues predicted for coordinated myristoylation and palmitoylation. Surprisingly, we show that disruption of ISP1 results in a dramatic relocalization of ISP2 and ISP3 to the apical cap. Although the N-terminal region of ISP1 is necessary and sufficient for apical cap targeting, exclusion of other family members requires the remaining C-terminal region of the protein. This gate-keeping function of ISP1 reveals an unprecedented mechanism of interactive and hierarchical targeting of proteins to establish these unique sub-compartments in the *Toxoplasma* IMC. Finally, we show that loss of ISP2 results in severe defects in daughter cell formation during endodyogeny, indicating a role for the ISP proteins in coordinating this unique process of *Toxoplasma* replication.

## Author Summary

Apicomplexans are the cause of important diseases in humans and animals including malaria (*Plasmodium falciparum*), which claims over a million human lives each year, and toxoplasmosis (*Toxoplasma gondii*), which causes birth defects and neurological disorders. These parasites possess a unique cortical system of membrane sacs arranged on a cytoskeletal meshwork, together referred to as the inner membrane complex (IMC). The IMC is the anchor point for the gliding motility machinery necessary for host invasion and also a scaffold around which new parasites are constructed during replication. Here we have uncovered new insights into the organization and function of this structure by identifying and characterizing ISP1-3, a family of proteins that define novel sub-compartments within the *Toxoplasma* IMC. Residues predicted for myristoylation and palmitoylation are critical in the membrane targeting of these proteins, suggesting that multiple palmitoyl acyltransferase activities reside within the IMC and dictate its organization. Surprisingly, ISP1 is required for proper sub-compartment sorting of ISP2 and 3, revealing a novel hierarchical targeting mechanism for the organization of this membrane system. Disruption of ISP2 results in defects during endodyogeny and a dramatic loss in parasite fitness, revealing that the ISP proteins play an important role in coordinating parasite replication.

## Introduction

The phylum Apicomplexa contains numerous obligate intracellular pathogens that are the cause of serious disease in humans and animals, greatly influencing global health and causing significant economic loss worldwide. The phylum includes *Plasmodium falciparum*, the causative agent of malaria which claims 1–2 million human lives annually, and *Toxoplasma gondii*, a pathogen that infects more than thirty percent of the world's population and causes severe neurological disorders and death in immunocompromised individuals [1]. Most of the drugs used to treat apicomplexans target metabolic pathways or the chloroplast-derived apicoplast [2], [3], [4], but these parasites also possess elaborate and unique structures that are required for replication and invasion and thus represent attractive new targets for therapeutic intervention.

Apicomplexans are grouped with dinoflagellates and ciliates in the alveolata infrakingdom [5]. The unifying morphological characteristic of this group is the presence of alveoli: membrane sacs located beneath the plasma membrane. Molecular phylogenetic data supports this grouping, as does the identification of a conserved family of articulin-like membrane skeleton proteins, the alveolins, which associate with alveoli in all three phyla [6], [7]. While the presence of alveoli is conserved, each of these groups has adapted this peripheral membrane structure for different cellular functions to fit their distinct niches. In dinoflagellates, the alveoli sometimes contain cellulose-based plates that function as protective armor [8]. In contrast, ciliate alveoli are calcium storage devices thought to play roles in regulation of cilia, exocytosis from cortical organelles known as extrusomes, and control of cytoskeletal elements [9], [10], [11].

In apicomplexans, the alveoli in conjunction with an underlying filamentous network are termed the inner membrane complex (IMC) [12], [13]. Flattened alveoli underlie the entirety of the plasma membrane except for a small gap at the apex and base of the cell [14]. These cisternae are organized into a patchwork of rectangular plates capped by a single cone-shaped plate at the apex of the cell. Freeze-fracture studies of the IMC plates expose a lattice of intramembranous particles (IMPs), an arrangement that suggests an association with proteins of the underlying filamentous network and subtending cortical microtubules [15], [16], [17]. Together, these features of the IMC are the foundation for a unique form of gliding motility used for host cell invasion and also serve as the scaffold for daughter cell formation during division [18], [19].

*Toxoplasma* tachyzoites replicate by endodyogeny, a process of internal cell budding that produces two daughters within an intact mother parasite. Following centriole duplication, daughter cell formation begins with the concurrent assembly of an apical and basal complex [20]. Although these two structures consist of cytoskeletal components that will eventually cap opposite ends of the mature parasite, they are initiated in close spatial and temporal proximity. IMC construction then proceeds by the extension of the basal complex away from the daughter apical complex, generating a bud into which replicated organelles are packaged. Parasite division is completed by a number of maturation steps terminating with the adoption of the maternal plasma membrane [21].

The apical, cone-shaped cisterna is unique in form and presumably the earliest membrane component deposited into the nascent IMC [19]. A number of cytoskeletal IMC markers localize to a region at the parasite apex thought to correspond to this apical-most IMC plate. A GFP fusion of the dynein light chain, TgDLC, can be detected in an apical cap region but predominantly localizes to the conoid and is also found in the basal complex, spindle poles and centrioles. TgCentrin2, the most divergent of the three *Toxoplasma* centrin homologues, labels the preconoidal rings and a peripheral ring of ∼6 annuli located at the lower boundary of the TgDLC cap. It has been suggested that these annuli lie at the juncture between the apical cap plate and the flanking set of IMC plates [20]. Additionally, PhIL1, a cytoskeletal IMC protein of unknown function, is detected throughout the IMC but strongly enriched in the apical cap and basal complex [22]. Only a few proteins are known to directly associate with the IMC membranes. These include a number of proteins associated with gliding motility [23], [24], [25], as well as the heat shock protein Hsp20 [26] and one isoform of the purine salvage enzyme hypoxanthine-xanthine-guanine phosphoribosyltransferase [27]. Thus, despite the central role of this conserved membrane system in apicomplexan biology, little is known of its composition, organization, and construction.

We present here a family of proteins unique to the Apicomplexa that localize to three distinct sub-compartments of the *Toxoplasma* IMC. ISP1 localizes to a region corresponding to the apical cap, ISP2 occupies a central IMC region, and ISP3 resides in both the central IMC region and a basal IMC compartment. ISP1 and 3 are early markers for bud formation and label previously unobserved daughter IMC structures in the absence of parasite cortical microtubules, indicating that microtubules are not required for initial assembly of IMC membranes. We show that the ISPs are initially targeted to the IMC by conserved residues predicted for coordinated myristoylation and palmitoylation in the extreme N-terminus of each of these proteins. Interestingly, deletion of *ISP1* results in the relocalization of ISP2 and 3 to the apical cap, demonstrating an interactive, hierarchical targeting among this family of proteins to these distinct sub-compartments of the IMC. Finally, disruption of *ISP2* results in a severe loss of parasite fitness and dramatic defects in daughter cell formation. Although the ISP2 knockout parasites ultimately compensate for these defects, this data shows an important role for these proteins in the coordination of daughter cell assembly.

## Results

### Monoclonal antibody 7E8 labels the apical cap of *Toxoplasma*

**Figure 1:**
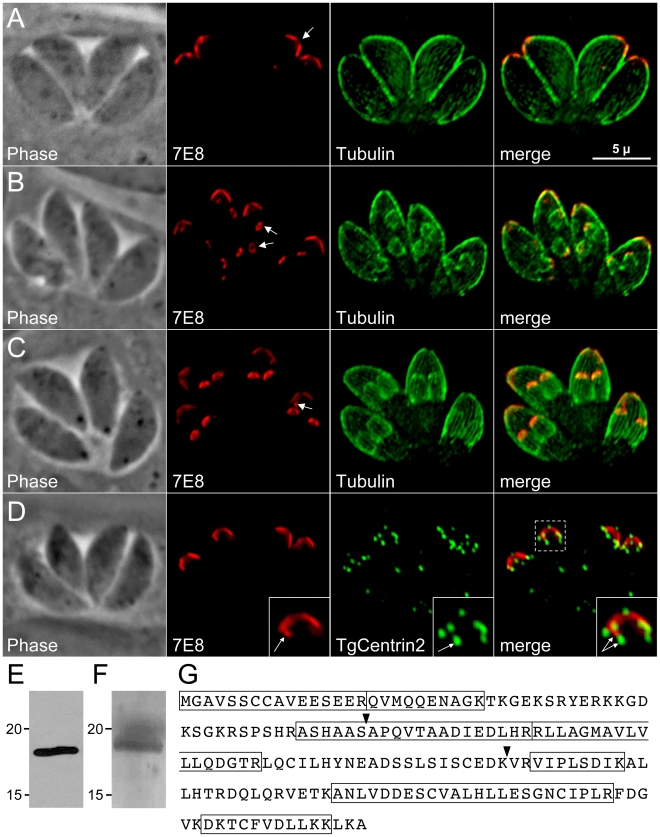
mAb 7E8 stains an apical cap structure in mature *Toxoplasma* tachyzoites and forming daughter parasites. **A**–**C.** IFA labeling with 7E8 and anti-tubulin displaying parasites before the onset of endodyogeny (A), early in endodyogeny (B), and late in endodyogeny (C). (A) 7E8 labels a peripheral cone-shaped structure at the parasite apex ∼1.5 µm in length. A gap in staining exists at the apex of the cone (arrow). (B) During early endodyogeny, 7E8 labels small rings with a central hole at the apex of forming daughter parasites (arrows). (C) As endodyogeny proceeds, the 7E8 rings enlarge and elongate into the apical cap structures seen in mature tachyzoites. 7E8 also labels a single spot in many parasites, which resides near the base of the apical cap but is clearly distinct (arrow). This spot is present through the cell cycle as seen in (A) and (B). We denote it here in late endodyogeny to demonstrate that it is a distinct structure and not merely an early daughter bud. Red: mAb 7E8 detected by Alexa594-anti-mouse IgG. Green: anti-tubulin antibody detected by Alexa488-anti-rabbit IgG. Scale bar  = 5 µm. **D.** 7E8 staining is delimited at both its apex and base by TgCentrin2, which labels the preconoidal rings as well as a series of annuli further down the cell periphery in the apical end of the parasite. The 7E8 apical spot does not colocalize with TgCentrin2 annuli (inset arrows). TgCentrin2 also localizes to the centriole and the basal complex. Red: 7E8 antibody detected by Alexa488-anti-mouse IgG (pseudo-colored red for consistency in the color scheme). Green: mRFP-TgCentrin2 (pseudo-colored green). **E.** Western blot analysis of *Toxoplasma* lysates by 7E8 detects a single band at ∼18 kD. **F.** The 7E8 immunoaffinity purified 18 kD protein visualized by Coomassie-staining in an SDS-PAGE gel. The band was excised from the gel, digested by trypsin and the resulting peptide fragments identified by mass spectrometry. **G.** The 176 amino acid sequence of ISP1, the protein recognized by 7E8. Boxed regions indicated 7 tryptic peptides identified by MS/MS. Arrowheads denote exon boundaries.

We previously generated a panel of monoclonal antibodies against a mixed fraction of *T. gondii* organelles [28]. One of the antibodies, 7E8, stains a cone-shaped structure at the periphery of the apical end of the parasite (Figure 1A). This staining pattern extends from a gap at the extreme apex (Figure 1A, arrow) ∼1.5 µm along the length of the parasite, a localization suggestive of the apical IMC plate observed by electron microscopy [14]. Colocalization with TgCentrin2 shows that 7E8 staining is delimited at its apex and base by this apical cap marker, indicating that 7E8 does indeed detect a protein associated with the anterior-most IMC plate (Figure 1D).

During early endodyogeny, 7E8 staining is visible in daughter parasites as a pair of small rings within each mother parasite (Figure 1B, arrows). As daughter formation proceeds, this structure enlarges and extends to form the apical cap seen in mature tachyzoites (Figure 1C). The association with forming daughter scaffolds together with the extreme apical gap further suggests that 7E8 labels the apical sub-compartment of the IMC. We also frequently observe 7E8 staining a single dot near the basal border of the cone (Figure 1C, arrow) which is distinct from TgCentrin2 annuli (Figure 1D, inset).

### Identification of ISP1, the *Toxoplasma* protein recognized by mAb 7E8

Western blot analysis of *Toxoplasma* lysates with mAb 7E8 revealed a single band at ∼18 kDa (Figure 1E). We used the 7E8 antibody to isolate its target protein by immunoaffinity chromatography. The isolated protein was separated by SDS-PAGE (Figure 1F), digested with trypsin, and seven peptides were identified by mass spectrometry corresponding to the hypothetical *T. gondii* protein TGGT1_009340 (Figure 1G). EST and cDNA sequencing confirmed that the gene model is correct. Due to its unique localization, we named this protein IMC Sub-compartment Protein 1 (ISP1).

Examination of the 176 amino acid sequence of ISP1 reveals that it contains a high number of charged residues (∼30%). While there are a relatively large number of ESTs encoding ISP1, the protein lacks conserved domains that could suggest its function. The protein contains a glycine at position two, which is predicted to be myristoylated [29] as well as a pair of cysteines at positions seven and eight strongly predicted to be palmitoylated [30]. Since ISP1 lacks a predicted signal peptide or transmembrane domain, these residues suggested a mechanism for IMC membrane association. BLAST analysis of the ISP1 sequence revealed orthologues across the apicomplexan phylum, including *Neospora, Theileria, Cryptosporidia, Babesia,* and *Plasmodium* (Figure S1). Orthologues were also found in *Eimeria* by BLAST against EST libraries (data not shown). ISP1 also showed significant homology in its C-terminal region to CP15/60, a poorly characterized putative surface glycoprotein in *Cryptosporidia* [31], [32]. No ISP1 orthologues were identified outside of the phylum indicating that this protein is restricted to the Apicomplexa.

### Identification and localization of ISP2 and ISP3

**Figure 2:**
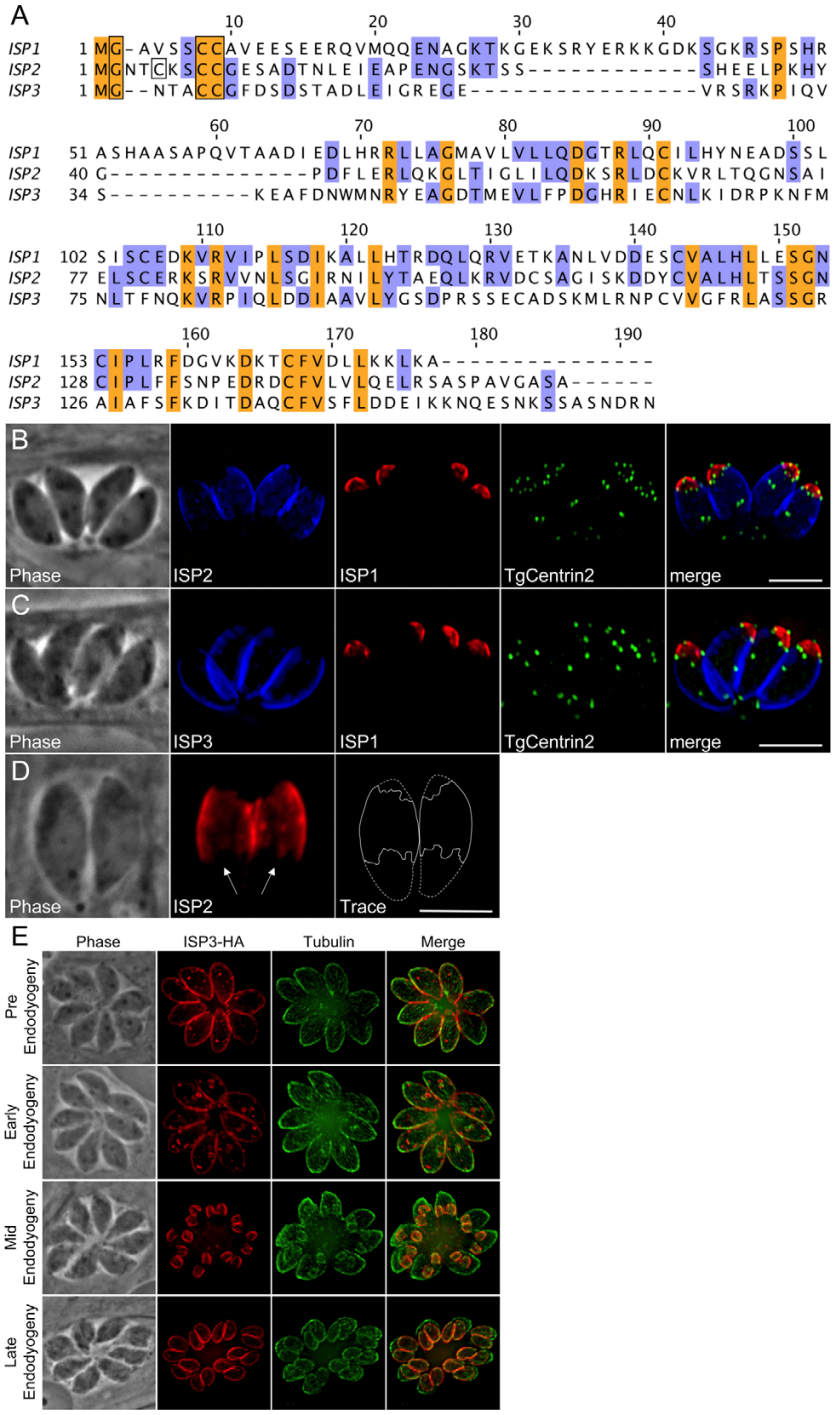
ISP2 and ISP3 define two additional novel sub-compartments of the IMC. **A.** BLAST analysis within the *T. gondii* genome identified two paralogs of ISP1 we denoted ISP2 and ISP3. The greatest sequence homology is present within the C-terminal portion of this family of proteins beginning at residue 71 of ISP1 while the N-terminal portion of each protein is more divergent. ISP1 and ISP2 show a higher sequence similarity with each other compared to ISP3. All family members have a conserved glycine at position two predicted to be myristoylated and a pair of conserved cysteines predicted to be palmitoylated within the first 10 residues (boxed). ISP2 contains an additional cysteine at position 5 that is predicted to be palmitoylated (boxed). The gene models for ISP1-3 were confirmed by cDNA sequencing. Nucleotide sequences are available in GenBank under the accession numbers HQ012577-HQ012579. **B**–**C.** ISP2 and ISP3 were expressed with a C-terminal HA epitope-tag under the control of their endogenous promoter in parasites expressing mRFP-TgCentrin2. (B) ISP2 localizes to a novel, peripheral sub-compartment beginning at the basal border of the ISP1 apical cap and extending approximately two-thirds the length of the parasite. ISP2 is not found in the basal third of the parasite periphery. (C) ISP3 staining overlaps with ISP2 but extends further to the base of the parasite where there is a small gap in staining, indicating an association with the IMC. The boundary between the ISP1 apical cap and the sub-compartments labeled by ISP2 and ISP3 is occupied by a ring of TgCentrin2 annuli. Blue: anti-HA antibody detected by Alexa350-anti-rabbit IgG. Red: 7E8 antibody detected by Alexa488-anti-mouse IgG (pseudo-colored red). Green: mRFP-TgCentrin2 (pseudo-colored green). **D.** The base of the ISP2 compartment terminates in a jagged edge (arrows). A trace of the ISP2 compartment boundary (solid line) was performed to illustrate this feature within the whole parasite (dashed line). Red: anti-HA antibody detected by Alexa594-anti-mouse IgG. **E.** Different stages of daughter budding were observed in parasites stably expressing ISP3-HA. With the beginning of endodyogeny, the maternal ISP3 signal decreases as the signal increases in daughter parasites. By mid-endodyogeny, ISP3 has disappeared completely from the maternal cell periphery. The parasites used in these images are *Δisp1*, thus ISP3 targets throughout the IMC, including the apical cap (see Figure 7). Red: anti-HA antibody detected by Alexa594-anti-mouse IgG. Green: anti-tubulin antibody detected by Alexa488-anti-rabbit IgG. All scale bars  = 5 µm.

BLAST analysis of the *T. gondii* genome using the ISP1 sequence identified two additional hypothetical proteins with considerable sequence similarity to ISP1, which we named ISP2 (TGGT1_058450) and ISP3 (TGGT1_094350) (Figure 2A). The greatest degree of sequence similarity between these three proteins exists within the C-terminal two-thirds of their sequences. The N-terminal regions of the proteins are more divergent, but each contain a conserved glycine at position two as well as a pair of conserved cysteines predicted to be myristoylated and palmitoylated, respectively (Figure 2A, boxed residues). ISP2 additionally contains a third cysteine at position five predicted to be palmitoylated. Similar to ISP1, these proteins are highly charged and have a relatively large number of corresponding ESTs. OrthoMCL analysis of the ISPs indicates two ortholog groups within Apicomplexa. ISP1 and ISP2 segregate with one group while ISP3 segregates with another (Figure S1). The *Toxoplasma* genome may encode a fourth ISP family member (TGGT1_063420), although it does not segregate with any OrthoMCL group. This predicted protein lacks the conserved glycine and cysteine residues present in the N-termini of other ISP proteins. Only a single EST is present for TGGT1_063420, indicating that it is poorly expressed relative to the other ISPs, and thus it was not investigated further.

To localize ISP2 and ISP3 in *T. gondii*, we expressed each gene under the control of its endogenous promoter with a C-terminal HA epitope tag. Intriguingly, ISP2 localizes to a previously unrecognized central sub-compartment of the IMC, which begins at the base of the ISP1 apical cap and extends approximately two-thirds the length of the cell. The apical boundary of this compartment is delineated by the TgCentrin2 annuli (Figure 2B). The posterior boundary has a jagged edge suggesting it corresponds to discrete IMC plates (Figure 2D, arrows). While the ISP2 signal terminates near the end of the subpellicular microtubules, the termini for these two structures are not identical (Figure S3, WT). Antisera raised against recombinant ISP2 confirmed this central IMC sub-compartment localization, ensuring that exclusion of ISP2 from the apical cap and basal IMC is not an artifact of epitope tagging (Figure S2A).

Similar to ISP2, ISP3 stains the central section of the IMC. However, ISP3 staining extends to the posterior end of the complex, identifying a third sub-compartment of the IMC (Figure 2C). A small gap in ISP3 staining is observed in the posterior region similar to that seen for other IMC proteins [23]. Antisera raised against recombinant ISP3 gave a poor signal by IFA, but was sufficient to confirm localization to both the IMC central and basal sub-compartments (Figure S2B). As with ISP1, ISP2 and ISP3 are visible in forming daughter parasites. Whereas the maternal signals of ISP1 and ISP2 appear to remain stable throughout endodyogeny, the maternal ISP3 signal rapidly attenuates with the onset of endodyogeny while it concentrates in daughters (Figure 2E). Attenuation of ISP3 in mothers and enrichment in daughters was also observed with our polyclonal antibody, indicating this is not the result of a C-terminal processing event that removes the HA epitope tag (Figure S2C). Thus, ISP3 provides an excellent marker for bud initiation, growth, and maturation during endodyogeny (Figure 2E and Video S1).

### The ISPs are associated with the IMC at the cell periphery but are not embedded in the IMC protein meshwork

**Figure 3:**
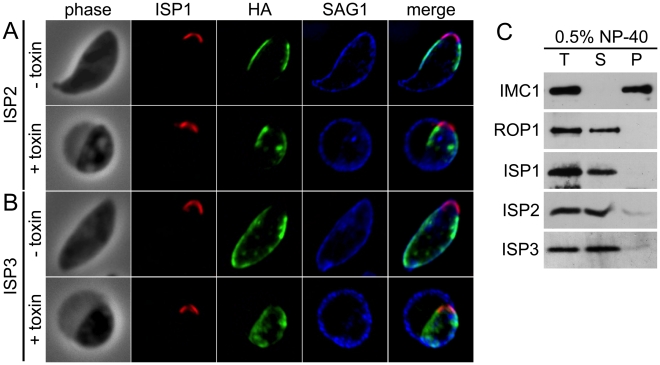
ISPs associate with the IMC but are not imbedded in the underlying protein meshwork. **A**–**B.** Extracellular parasites expressing ISP2-HA (A) or ISP3-HA (B) were incubated 4 hrs with or without 20 nM *Clostridum septicum* alpha-toxin. In untreated cells, the plasma membrane marker SAG1 cannot be resolved from the IMC membranes. Following alpha-toxin treatment, a dramatic swelling of the plasma membrane occurs, separating it from the underlying IMC and enabling resolution of these two membrane systems. Each ISP family member clearly segregates with the cell body and not the distended plasma membrane, indicating an association with the IMC. Red: 7E8 antibody detected by Alexa594-anti-mouse IgG. Green: anti-HA antibody detected by Alexa488-anti-rat IgG. Blue: anti-SAG1 antibody detected by Alexa350-anti-rabbit IgG. **C.** Parasites were extracted with 0.5% NP-40 and separated into total (T), soluble (S) and pellet (P) fractions. Extracts were subjected to SDS-PAGE, blotted and probed with antibodies as indicated. As expected, the detergent resistant IMC protein meshwork containing IMC1 remains in the pellet under these conditions. In contrast, ISP1-3 are resolved into the soluble fraction, similar to the soluble control protein ROP1, demonstrating that these proteins are not embedded in the protein meshwork of the IMC.

The observations that the ISPs are visible at the periphery of forming daughters prior to adoption of the maternal plasma membrane and that gaps are present at the extreme apex and base suggests an association with the IMC. To confirm IMC association, we treated extracellular parasites with *Clostridium septicum* alpha-toxin. This vacuolating toxin causes a dramatic separation of the plasma membrane and the underlying IMC, enabling differential localization of these closely apposed membrane systems [33]. In toxin-treated parasites, the ISP proteins segregate with the IMC and not with the plasma membrane, confirming that the ISPs are indeed IMC proteins (Figure 3A–B).

To ascertain if the ISPs are embedded in the IMC protein meshwork that includes the articulin-like protein IMC1, we performed detergent extractions of extracellular parasites in 0.5% NP-40. In these conditions, each ISP was solubilized similar to the control protein ROP1, while IMC1 remained in the insoluble pellet fraction (Figure 3C). This extraction profile demonstrates that the ISPs are not embedded in the detergent resistant protein meshwork that underlies the IMC membranes.

### ISP1 and ISP3 localize to nascent daughter buds in the absence of microtubules

**Figure 4:**
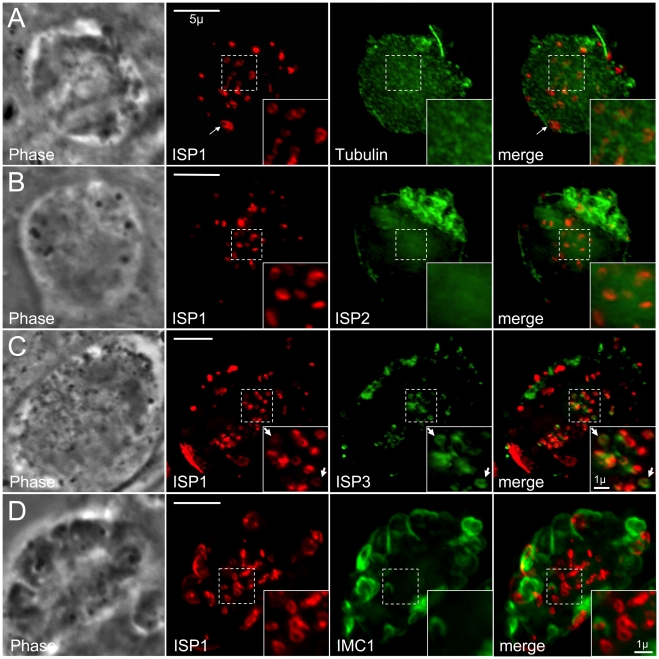
ISP1 and ISP3 are targeted to early daughter buds in the absence of parasite microtubules. **A.** Parasites were treated with 2.5 µM oryzalin for 40 hrs. In the absence of microtubules, ISP1 labels numerous ring structures (inset) within the center of the cell reminiscent of early daughter buds. Unpolymerized parasite tubulin is dispersed throughout the cytoplasm. Red: mAb 7E8 detected by Alexa594-anti-mouse IgG. Green: YFP-αTubulin. **B.** Parasites expressing ISP2-HA were treated with 2.5 µM oryzalin for 40 hrs. ISP2 is localized in patches at the cell periphery and does not appear to associate with the ISP1 labeled rings (inset). Red: mAb 7E8 detected by Alexa594-anti-mouse IgG. Green: anti-HA antibody detected by Alexa488-anti-rabbit IgG. **C.** Parasites expressing ISP3-HA were treated with 2.5 µM oryzalin for 40 hrs. ISP3 is also localized to the ring structures labeled by ISP1 (inset arrows), although ISP1 and 3 rings do not always perfectly colocalize. Red: mAb 7E8 detected by Alexa594-anti-mouse IgG. Green: anti-HA antibody detected by Alexa488-anti-rabbit IgG. **D.** Parasites expressing ISP1-HA were treated with 0.5 µM oryzalin for 30 hrs. IMC1 labels partially formed parasites and sheets of IMC1 at the cell periphery and ISP1 apical cap staining can be observed at the apex of some of these structures. However, the majority of ISP1 signal is still localized to rings within the center of the cell under these less stringent conditions. These rings do not associate with IMC1 stained structures (insets). A 3D projection of this image is presented in Video S2. Red: anti-HA antibody detected by Alexa594-anti-rabbit IgG. Green: anti-IMC1 antibody detected by Alexa488-anti-mouse IgG. Scale bars  = 5 µm. Inset scale bars  = 1 µm.

We disrupted microtubules in intracellular parasites to assess whether the underlying microtubules influence ISP localization. Apicomplexan microtubules are selectively susceptible to disruption by dinitroanilines, such as oryzalin [34]. After 40 hours of 2.5 µM oryzalin treatment, all tubulin is unpolymerized and dispersed. Without spindle microtubules (mitosis) and subpellicular microtubules (budding), productive daughter formation repeatedly fails resulting in an undivided, amorphous mother cell with a polyploid DNA content [35] (Figure 4A). Intriguingly, we observe ISP1 labeling numerous small rings that are centrally located within oryzalin-treated parasites (Figure 4A, inset) of approximately the same dimensions as ISP1 early daughter buds in untreated, replicating parasites (compare with Figure 1B, arrows). Since polymerization of subpellicular microtubules is essential to drive bud extension, these rings likely represent failed attempts to build new daughter buds [36]. A larger peripheral patch of ISP1 with a central hole is also observed, likely representing the original parent apical cap (Figure 4A, arrows). While ISP2 was not observable in these early bud rings (Figure 4B), we did detect ISP3 in these structures within oryzalin-treated parasites (Figure 4C, inset arrows), suggesting that both the apical cap and remaining IMC sub-domains are formed independently of microtubules at a very early stage of bud development. While membrane skeleton proteins are likely candidates for providing the foundation for these structures, we were unable to detect the articulin-like protein IMC1 in these early bud rings, even at lower oryzalin concentrations (0.5 µM) that only disrupt cortical microtubules (Figure 4D and Video S2).

### An N-terminal region is sufficient for sub-compartment targeting of ISP1 and ISP3 but not ISP2

**Figure 5:**
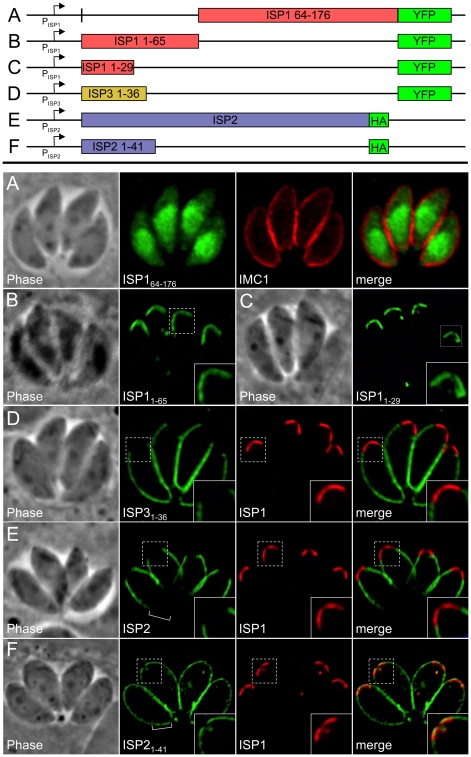
An N-terminal domain is sufficient for IMC sub-compartment targeting of ISP1 and 3 but not ISP2. **A.** The C-terminal portion of ISP1 (residues 64–176), which bears the greatest sequence homology with other ISP family members, was expressed with a C-terminal YFP tag. The ISP1_64–176_-YFP protein is dispersed throughout the cytosol and nucleus, demonstrating that the first 63 residues of ISP1 are necessary for IMC apical cap targeting. Green: ISP1_64–176_-YFP. Red: anti-IMC1 antibody detected by Alexa594-anti-mouse IgG. **B**–**C.** Residues 1–65 (containing the putative acylation sequence and divergent N-terminal region) or 1–29 (containing the putative acylation sequence) of ISP1 were expressed with a C-terminal YFP fusion. Both proteins target in an identical manner to endogenous ISP1, demonstrating that the first 29 residues are sufficient for IMC apical cap targeting (cap shown in inset). Green: ISP1_1–65_-YFP or ISP1_1–29_-YFP. **D.** Residues 1–36 of ISP3 were expressed with a C-terminal YFP fusion. The ISP3_1–36_-YFP protein targets in an identical manner to endogenous ISP3, including exclusion from the apical cap demonstrated by non-overlapping signal with ISP1 (inset), showing that these residues are sufficient for proper ISP3 sub-compartment targeting within the IMC. Green: ISP3_1–36_-YFP. Red: mAb 7E8 detected by Alexa594-anti-mouse IgG. **E.** Targeting of full length ISP2-HA is restricted to the central IMC sub-compartment identical to endogenous ISP2 as shown by non-overlapping signal with ISP1 in the apical cap (inset) and lack of signal in the basal IMC sub-compartment (bracket). **F.** Residues 1–41 of ISP2 were expressed with a C-terminal HA tag. The ISP2_1–41_-HA protein targets to all three sub-compartments of the IMC, as shown by overlap with endogenous ISP1 in the apical cap (inset) and signal within the basal IMC sub-compartment (bracket). A small gap is visible at the extreme apex and base of the ISP2_1–41_-HA staining, indicating this protein is still targeting to the IMC. Identical results were seen using YFP in place of HA (data not shown). Green: anti-HA antibody detected by Alexa488-anti-rabbit IgG. Red: mAb 7E8 detected by Alexa594-anti-mouse IgG.

The greatest sequence similarity within the ISP family is present in the C-terminal two-thirds of the proteins while the N-terminal region is more divergent (Figure 2A), thus we reasoned that the unique targeting of each ISP family member might be controlled by its N-terminal region. To test if the N-terminal region of ISP1 is necessary for targeting, we eliminated the first 63 residues to create a truncated protein fused to YFP. ISP1_64–176_-YFP does not target to the IMC but is instead distributed throughout the cytoplasm and nucleus, showing that this N-terminal region is necessary for apical cap targeting (Figure 5A). To determine if the ISP1 N-terminal region is sufficient for targeting, we fused the first 65 residues of ISP1 (containing the putative acylation sequence and divergent N-terminal region) to YFP and expressed this construct in *Toxoplasma*. The ISP1_1–65_-YFP fusion traffics to the apical cap in an identical fashion to endogenous ISP1 (Figure 5B). To further narrow the N-terminal region required for apical cap targeting, we generated an additional fusion of the first 29 residues of ISP1 (containing the putative acylation sequence) to YFP. This fusion also traffics in a manner identical to full length ISP1 (Figure 5C), demonstrating that this N-terminal domain is both necessary and sufficient for apical cap targeting.

To assess targeting of ISP2 and ISP3, we also created fusions of their N-terminal regions (residues 1–41 and 1–36 respectively) to YFP. The ISP3_1–36_-YFP fusion targets to the central and basal sub-compartments of the IMC but is restricted from the apical cap (Figure 5D), showing that this region is sufficient for proper sub-compartment targeting. In contrast, ISP2_1–41_-YFP localized to the entire IMC, overlapping with endogenous ISP1 in the apical cap and extending into the basal IMC sub-compartment (data not shown). To ensure this change in targeting for ISP2_1–41_ was not an artifact of the YFP fusion, we replaced YFP with an HA tag (shown to have no effect on the targeting of full length ISP2, Figure 5E). The ISP2_1–41_-HA protein also localized throughout the IMC (Figure 5F), demonstrating that the N-terminal domain of ISP2 is sufficient for targeting to the IMC, but not for correct sub-compartment localization.

### Conserved N-terminal residues predicted for acylation are critical for ISP targeting

**Figure 6:**
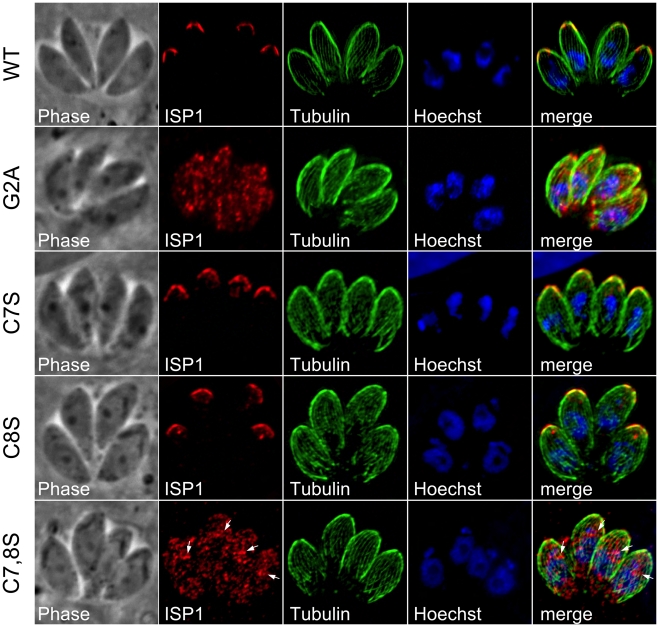
Mutation of ISP1 residues predicted for acylation results in ISP1 mistargeting. Mutations of residues predicted for myristoylation or palmitoylation were generated in an HA epitope-tagged copy of ISP1 and expressed in parasites under the endogenous promoter. Wild-type (WT) ISP1-HA targets in an identical fashion to endogenous ISP1. A severe targeting defect occurs in ISP1(G2A) with the mutant protein dispersed throughout the cell in a punctate fashion. Mutation of individual cysteines predicted for palmitoylation (C7S and C8S) produces no significant defect in targeting, but coordinated mutation of these cysteines results in gross mistargeting of ISP1(C7,8S) throughout the cell in a punctate fashion with a signal accumulation just apical of the nucleus (arrows). Red: anti-HA antibody detected by Alexa594-anti-mouse IgG. Green: anti-tubulin antibody detected by Alexa488-anti-rabbit IgG. Blue: Hoechst stain.

Protein myristoylation occurs co-translationally through the action of an N-myristoyl transferase [37]. This modification is sufficient to promote transient association with membranes for otherwise cytosolic proteins. This weak membrane affinity can then be stabilized by addition of one or more palmitoylations through the action of a palmitoyl acyltransferase (PAT), effectively locking a protein into a target membrane system in a mechanism known as “kinetic trapping”. The ISPs each contain a second position glycine followed by cysteines within the first 10 residues that are predicted to be myristoylated and palmitoylated, respectively (Figure 2, boxed residues). We mutated the glycine and cysteine residues in HA epitope tagged ISP constructs to examine their effect on targeting. As predicted by the kinetic trapping model, mutation of the second position glycine to an alanine abolished IMC targeting in each family member (Figure 6 and Figures S3 and S4, G2A), resulting in proteins distributed throughout the cytoplasm. Mutation of the cysteine residues to serine was performed individually and together. While only minor defects in targeting were observed with individual cysteine mutations, mutation of both cysteines abolished ISP1 and ISP3 targeting (Figure 6 and Figure S4). In the case of ISP2, targeting was only abolished when all three cysteines were coordinately mutated (Figure S3). While coordinated cysteine mutants of the ISPs are distributed in the cytoplasm similar to G2A mutants, we also often observed perinuclear staining that is especially concentrated just apical of the nucleus (arrows, Figure 6 and Figure S3 and S4). Presumably, myristoylation of these proteins still occurs, but without palmitoylation, these mutants are left to transiently sample the different membrane systems within the cell and therefore may appear concentrated as they associate with the ER and Golgi membranes present in this region. These results demonstrate that these residues are essential to ISP sorting and indicate that coordinated acylation of the ISPs is responsible for IMC membrane targeting.

### Disruption of ISP1 results in relocalization of ISP2 and ISP3 to the apical cap

**Figure 7:**
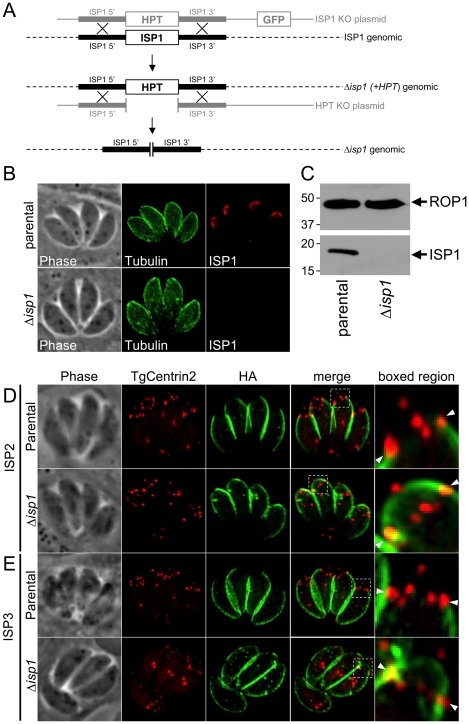
ISP2 and ISP3 are relocalized to the apical cap in the absence of ISP1. **A.** Schematic showing the *ISP1* knockout strategy. Double homologous recombination results in the replacement of *ISP*1 with the selectable marker *HPT* and the loss of the downstream marker *GFP*. An additional round of homologous recombination removes *HPT* to exclude any polar or selectable marker effects. **B.** Loss of ISP1 is demonstrated by the absence of 7E8 staining by IFA in *Δisp1* parasites. Red: mAb 7E8 detected by Alexa594-anti-mouse IgG. Green: anti-tubulin antibody detected by Alexa488-anti-rabbit IgG. **C.** Western blot analysis detects ISP1 in parental strain but not in *Δisp1* parasites. ROP1 serves as a loading control. **D.** ISP2 localization in wild-type parasites is non-overlapping with ISP1 and ends sharply at the basal boundary of the apical cap normally occupied by ISP1. A ring of TgCentrin2 annuli resides at this boundary (arrowheads). In *Δisp1* parasites, ISP2 relocalizes above the TgCentrin2 boundary, filling the apical cap. **E.** ISP3 is also relocalized to the apical cap in *Δisp1* parasites as assessed by the co-marker TgCentrin2. Red: mRFP-TgCentrin2. Green: anti-HA antibody detected by Alexa488-anti-rabbit IgG.

**Figure 8:**
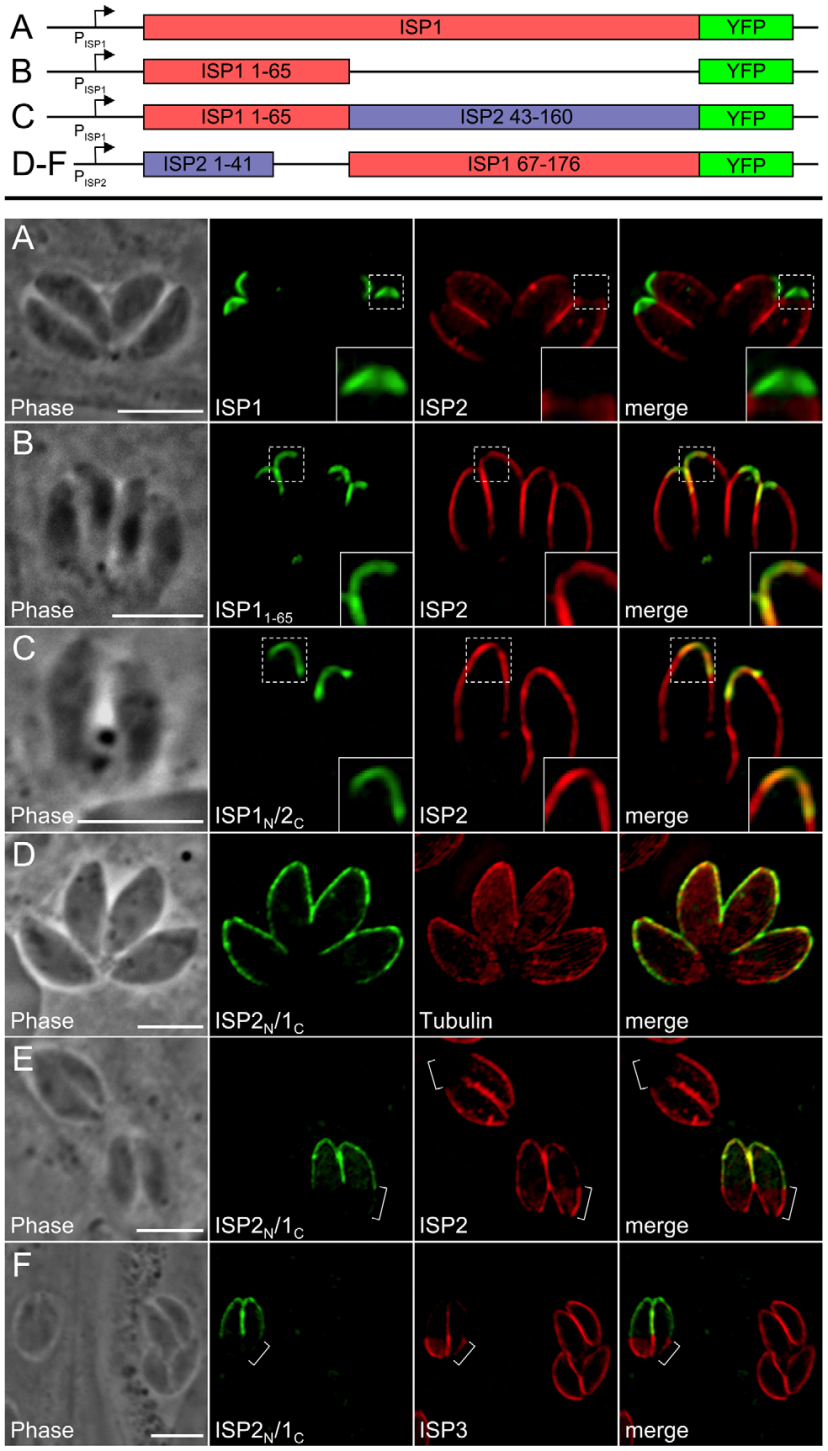
Exclusion of ISP2 and ISP3 is mediated by the C-terminal domain of ISP1. **A.** Full length ISP1 with a C-terminal YFP fusion was expressed in *Δisp1* parasites together with ISP2-HA. ISP1-YFP targets correctly to the apical cap and reestablishes normal localization of ISP2, excluding it from this region of the IMC (insets). Green: ISP1-YFP. Red: anti-HA antibody detected by Alexa594-anti-mouse IgG. **B.** ISP1_1–65_-YFP was expressed in *Δisp1* parasites together with ISP2-HA. While ISP1_1–65_-YFP targets correctly, ISP2 continues to relocalize to the apical cap in the presence of this truncated ISP1 protein (insets), demonstrating that the ISP1 C-terminal region (residues 66–176) is necessary for exclusion of ISP2 from the apical cap. Green: ISP1_1–65_-YFP fusion. Red: anti-HA antibody detected by Alexa594-anti-mouse IgG. **C.** A chimeric protein consisting of the ISP1 N-terminus (residues 1–65) and the ISP2 C-terminus (residues 43–160) fused to YFP was expressed in Δ*isp1* parasites together with ISP2-HA. This chimeric protein targets to the apical cap but does not prevent relocalization of ISP2-HA into the cap, as seen by the overlap of the two signals (insets). Green: ISP1_N_/2_C_-YFP. Red: anti-HA antibody detected by Alexa594-anti-mouse IgG. **D.** A chimeric protein consisting of the ISP2 N-terminus and the ISP1 C-terminus fused to YFP (ISP2_N_/1_C_-YFP) was expressed in wild-type parasites. This chimeric protein targets to the apical cap and central IMC compartments. Green: ISP2_N_/1_C_-YFP. Red: anti-tubulin antibody detected by Alexa594-anti-rabbit IgG. **E**–**F.** The exclusion activity of the ISP1 C-terminal domain against ISP2/3 can function in other IMC sub-compartments. (E) ISP2_N_/1_C_-YFP was transiently expressed in Δ*isp1* parasites stably expressing ISP2-HA. ISP2 is relocalized to the base sub-compartment of the IMC in parasites expressing this chimera. ISP2 is not present in the base IMC sub-compartment in parasites that are not expressing the chimeric protein (brackets). (F) ISP2_N_/1_C_-YFP was transiently expressed in Δ*isp1* parasites stably expressing ISP3-HA. In the presence of the chimeric protein, ISP3 is concentrated in the base sub-compartment of the IMC (brackets). ISP3 is evenly distributed throughout the IMC of parasites that are not expressing the chimeric protein. Green: ISP2_N_/1_C_-YFP. Red: anti-HA antibody detected by Alexa594-anti-mouse IgG. All scale bars  = 5 µm.

To assess the function of ISP1, we disrupted the *ISP1* gene by homologous recombination (Figure 7A). We identified clones which lacked ISP1 expression by IFA and Western blot (Figure 7B–C), indicating successful disruption of the *ISP1* locus and demonstrating that ISP1 is not necessary for *in vitro* propagation of *T. gondii*. Disruption of *ISP1* did not result in any gross defect in parasite growth. However, we were surprised to find that both ISP2 and ISP3 were relocalized in the Δ*isp1* strain. In the parental strain, ISP2 staining terminates sharply at the ring of TgCentrin2 annuli bordering the base of the apical cap (Figure 7D, arrowheads). However, in Δ*isp1* parasites, ISP2 staining extends past this border, relocalizing to the apical cap sub-compartment of the IMC (Figure 7D). Apical cap relocalization is also observed for ISP3 in the Δ*isp1* strain (Figure 7E). To ensure the ISP2 and ISP3 relocalization to the apical cap is truly a result of the absence of ISP1, we reintroduced the *ISP1* gene with a C-terminal YFP fusion into the Δ*isp1* strain. This fusion protein targets correctly to the apical cap and, importantly, reestablishes the wild-type localization of ISP2 (Figure 8A, insets) and ISP3 (data not shown), excluding them from the apical cap. Thus, ISP1 exhibits a gate-keeping effect on ISP2 and 3, preventing access to the apical cap and establishing a hierarchy of protein targeting among these IMC sub-compartments. To determine if ISP1 performs a broader scaffolding function within the apical cap, we evaluated the localization of TgDLC1 using a GFP fusion; however, we observed no change in the localization of this protein in the absence of ISP1 (data not shown).

### An ISP1 C-terminal domain is necessary for exclusion of ISP2 and ISP3 from the apical cap

Given the ability of ISP1 to exclude other family members from the apical cap, we exploited our ISP1_1–65_-YFP construct to determine whether or not the N-terminal region that is sufficient for apical cap targeting also plays a role in exclusion from this compartment. Expression of this construct in Δ*isp1* parasites does not result in exclusion of ISP2 (Figure 8B) or ISP3 (data not shown) from the apical cap, demonstrating that distal sequences present in the more conserved regions of ISP1 (residues 66–176) are necessary for exclusion. To further assess whether the C-terminal region from another ISP family member could substitute for the ISP1 C-terminal domain and function in exclusion, we constructed a hybrid protein containing the N-terminal 65 amino acids of ISP1 and the C-terminal region of ISP2 (residues 43–160) fused to YFP. Similar to the ISP1_1–65_-YFP construct, the ISP1_N_/2_C_-YFP chimera targets to the apical cap but does not exclude ISP2 (Figure 8C) or ISP3 (data not shown). These results demonstrate that the exclusion activity of the C-terminal region of ISP1 is specific to this family member and cannot be replaced by the complementary region from ISP2.

We created an additional chimera consisting of the N-terminal region of ISP2 (residues 1–41) fused to the C-terminal region of ISP1 (residues 67–176). While the N-terminal region of ISP2 alone targets YFP or HA throughout the IMC (Figure 5F), inclusion of the C-terminal region of ISP1 restricts the localization to the apical cap and central regions of the IMC (Figure 8D, see discussion). In parasites expressing this chimera, ISP2 and 3 are mostly relocalized into the base portion of the IMC (Figure 8E–F, brackets). The fact that the ISP1 C-terminal region is able to exhibit exclusion activity against the other ISPs when artificially targeted to other domains of the IMC strengthens the conclusion that the ISP1 C-terminal region constitutes an ISP exclusion domain.

### Disruption of ISP2 results in a severe loss of parasite fitness and division defects in daughter cell formation

**Figure 9:**
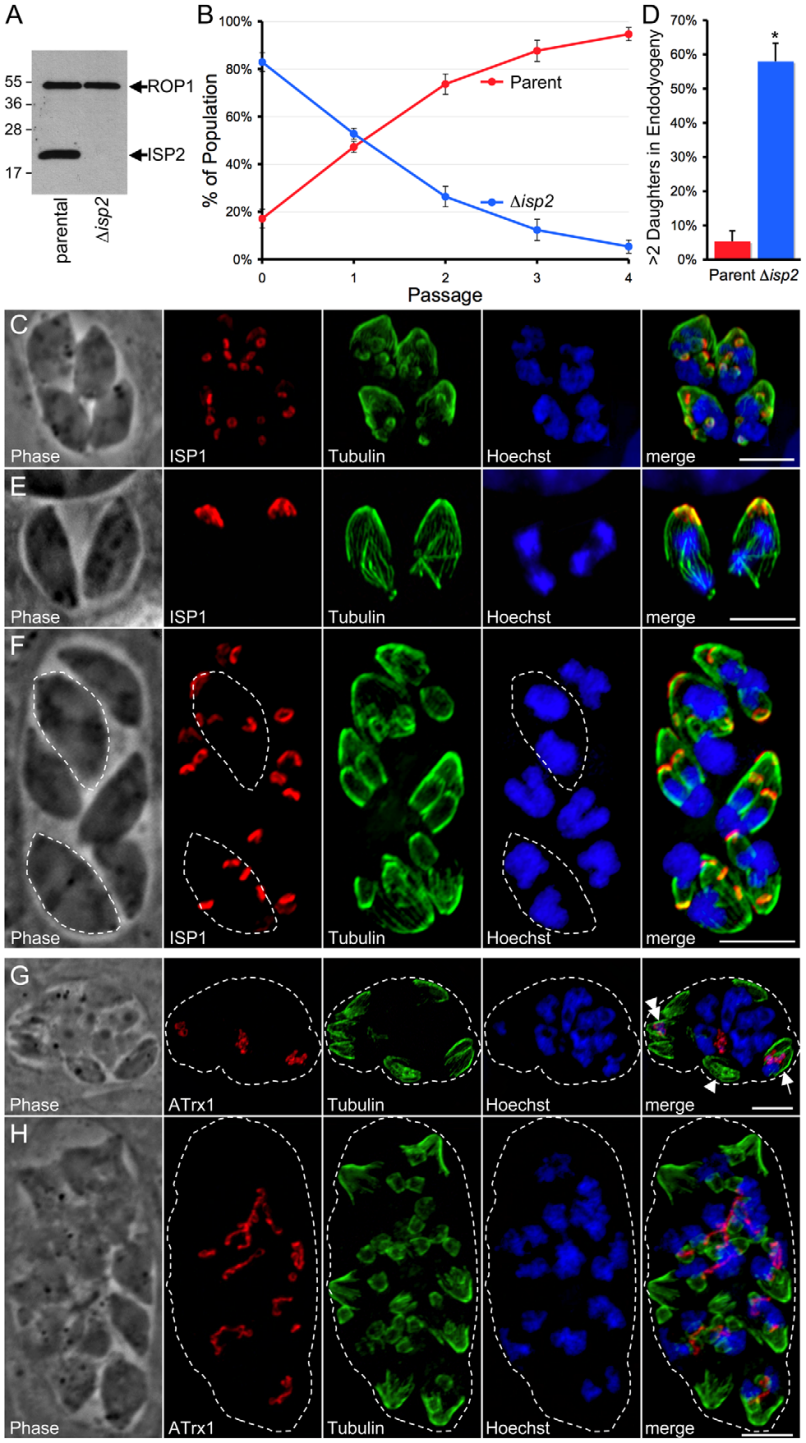
Disruption of ISP2 results in a severe loss of parasite fitness and defects in daughter cell formation. **A.** Western blot analysis using polyclonal anti-ISP2 confirms the loss of ISP2 in *Δisp2* parasites. ROP1 serves as a loading control. **B.** A competition growth assay reveals a severe fitness loss in Δ*isp2* parasites. Parent and Δ*isp2* parasites were mixed in culture and passaged. At each passage, the composition of the mixed culture was evaluated by IFA. Although Δ*isp2* parasites initially comprised >80% of the culture, they were rapidly out competed by the parental strain and essentially lost from the culture within four passages. Values represent means ± 3 standard deviations. **C.** Parasites lacking ISP2 assemble >2 daughters per round of endodyogeny. ISP1 was used as a marker for daughter buds. The top left parasite in this vacuole is assembling four daughters while the other three parasites are assembling five daughters each. While the top left parasite has divided its nucleus and is now budding two daughters around each of two separate nuclei, the other three parasites appear to each be budding five daughters around a single polyploid nucleus. Red: mAb 7E8 detected by Alexa594-anti-mouse IgG. Green: anti-tubulin antibody detected by Alexa488-anti-rabbit IgG. Blue: Hoechst stain. **D.** Quantification of the >2 daughters phenotype in Δ*isp2* parasites. Parasites undergoing endodyogeny were counted and scored for the percentage of vacuoles in which parasites were assembling >2 daughters. Most vacuoles contain one or more parasites assembling >2 daughters in the Δ*isp2* strain. Values represent means ± SD, n = 3, from a representative experiment. *P<0.001. **E**–**F.** Parasites lacking ISP2 can perform karyokinesis before budding. (E) ISP1 is an early marker for bud formation visible before nuclear segregation during endodyogeny. No daughter ISP1 signal is visible in these parasites although the spindle apparatus is assembled and has already separated the chromosomes into two nuclei, showing that karyokinesis can precede budding in Δ*isp2* parasites. (F) After undergoing a round of karyokinesis without budding, Δ*isp2* parasites can bud around each of the segregated nuclei. This vacuole contains two parasites (dashed outlines) that have undergone karyokinesis prior to budding and are now assembling two daughters around each individual nucleus. Red: mAb 7E8 detected by Alexa594-anti-mouse IgG. Green: anti-tubulin antibody detected by Alexa488-anti-rabbit IgG. Blue: Hoechst stain. **G**–**H.** Parasites lacking ISP2 display catastrophic replication defects. Parasites were stained for the apicoplast thioredoxin-like protein 1 (ATrx1), which labels the apicoplast, as well as tubulin and DNA. (G) In the vacuole shown, a few parasites have received both a nucleus and an apicoplast (arrow) while others contain only an apicoplast (double arrowhead) and some contain neither (arrowhead). Several nuclei have been extruded into the vacuole along with one or more apicoplasts. (H) Other vacuoles containing extruded nuclei also contained several daughter buds that appear to be outside of an intact mother parasites (∼18 in this vacuole visible by tubulin). Many nuclei and elongated apicoplasts are present in the vacuole and appear associated with the forming buds. For clarity, a dashed line indicates the boundary of the parasitophorus vacuole. Red: anti-ATrx1 detected by Alexa594-anti-mouse IgG. Green: anti-tubulin antibody detected by Alexa488-anti-rabbit IgG. Blue: Hoechst stain. All scale bars  = 5 µm.

To further investigate the function of the ISP proteins, we disrupted the genes encoding ISP2 and ISP3 by homologous recombination. To accomplish this, we employed a recently developed Δ*ku80* parasite strain that is highly efficient at homologous recombination [38]. We first removed *HPT* from the *Ku80* locus by homologous recombination and negative selection using 6-thioxanthine, creating Δ*ku80*Δ*hpt* strain parasites. We then used this strain to disrupt *ISP2* or *ISP3* and confirmed these deletions by IFA (not shown) and Western blot (Figure 9A and Figure S5).

In contrast to our findings for Δ*isp1* parasites, localization of other ISP family members was unchanged in both Δ*isp2* and Δ*isp3* strains (data not shown). While no gross phenotype was seen in Δ*isp3* parasites, the Δ*isp2* strain parasites were obviously defective in growth as the knockout was rapidly lost from transfected populations and its isolation required cloning early following transfection. To assess this loss in fitness, we performed competition growth assays between parent and Δ*isp2* parasites by mixing these strains in culture and monitoring the culture composition at each passage. The parental strain rapidly out competed the Δ*isp2* parasites, confirming a severe fitness loss in these parasites (Figure 9B). Further analysis by IFA revealed that Δ*isp2* parasites display a number of defects in parasite division. Most frequently, we observed the construction of >2 daughters per mother cell in each round of endodyogeny with some parasites assembling as many as 8 daughters (Figure 9C). To quantify this defect, we stained for ISP1, an early marker for bud formation during endodyogeny, and counted vacuoles containing parasites undergoing endodyogeny and assembling >2 buds. As expected, we saw a dramatic increase in the number of parasites producing more than two daughters in the Δ*isp2* strain (Figure 9D). Neither Δ*isp1* or Δ*isp3* parasites showed any aberration in daughter cell assembly compared to wild-type parasites (data not shown).

Assembly of >2 daughters in Δ*isp2* parasites sometimes occurred around a single polyploid nucleus with karyokinesis accompanying budding (bottom left parasite, Figure 9C) while other parasites assembled the spindle apparatus and underwent karyokinesis without budding, resulting in a mother parasite with two nuclei (Figure 9E). We also observe parasites containing two discrete nuclei in the process of budding >2 daughters (outlined parasites, Figure 9F).

Less frequently, we observed a catastrophic failure of Δ*isp2* parasites to appropriately segregate nuclei, resulting in anucleate zoids and nuclei extruded in the vacuole (Figure 9G). These vacuoles also show major defects in apicoplast segregation with a few cells receiving both a nucleus and an apicoplast while some received only an apicoplast and others received neither. Finally, some vacuoles with nuclear segregation defects contained many immature buds within the vacuole (Figure 9H). These buds appear to be outside of any intact parasite and it is unclear if they were initiated within a mother cell and then somehow liberated into the vacuolar space or if they were the result of a budding event that was initiated within the vacuolar space itself. In these vacuoles, several elongated apicoplasts are strung throughout the vacuolar space, associated with the extracellular buds and nuclei.

Surprisingly, the Δ*isp2* parasites recovered from both the fitness and replication defects after approximately two months of culture (data not shown), preventing complementation by genetic rescue. To ensure these phenotypes are specific to the disruption of *ISP2* and not the consequence of any off target effects, we generated a second independent Δ*isp2* line. This line displayed the same loss of fitness and cell division defects, indicating these phenotypes are specifically linked to disruption of the *ISP2* locus (data not shown).

## Discussion

### The IMC in apicomplexan parasites

**Figure 10:**
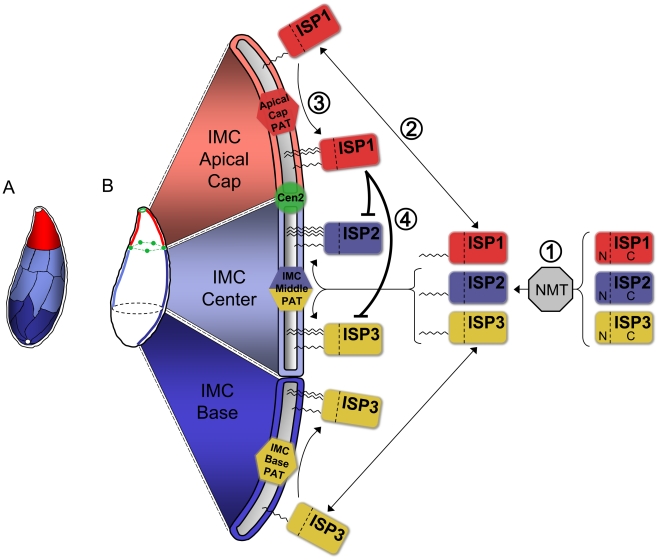
Model for ISP sorting within the parasite IMC. **A.** Alveoli, flattened membrane sacs, rest just under the plasma membrane atop a filamentous network in the *Toxoplasma* IMC. These alveoli are arranged as a patchwork of rectangular plates with a unique cone-shaped plate capping the apex. A small gap is present at the extreme apex and base of the IMC. The ISPs reveal three distinct sub-compartments within the IMC: the apical cap (red, ISP1), central IMC (light blue, ISP2 and ISP3), and basal IMC (dark blue, ISP3). Colors indicate speculative arrangement of sub-compartments within discrete alveoli or combinations thereof. **B.** Model for ISP sorting within the IMC. (1) ISP1/2/3 are co-translationally myristoylated within the cytosol at a conserved second position glycine by the action of an N-myristoyl transferase (NMT). (2) This initial acylation allows the ISPs to transiently associate with the various membrane systems within the cell, including the cisternae of the IMC. (3) Different PATs (or PAT activities) located within the three distinct sub-compartments of the IMC specifically recognize and palmitoylate their unique ISP substrates, locking them into the appropriate sub-compartment. An apical cap PAT with specificity for ISP1 locks it into the cap while a central IMC PAT is able to recognize and palmitoylate both ISP2 and ISP3 within the central IMC sub-compartment. ISP3 is stably localized to the IMC base sub-compartment by the action of a basal IMC PAT. (4) The presence of ISP1 in the apical cap provides an additional level of sorting by preventing the localization of ISP2 and 3 into this sub-compartment. While the N-terminus of ISP1 is sufficient for its sorting to the apical cap, the ISP1 C-terminal domain is required to prevent the localization of other ISP family members into the cap, possibly through the modulation of the apical cap PAT specificity for ISP2 and 3.

Alveoli are the unifying morphological feature among ciliates, dinoflagellates and apicomplexans where these unique membrane stacks have been adapted to suit these divergent organisms in vastly different niches. In apicomplexans, the membrane stacks (the IMC) have been exploited to provide unique and critical roles in parasite replication, motility and invasion. Freeze-fracture studies reveal a highly sophisticated arrangement of IMC plates with dissimilar organization of IMPs in the apical versus lower plates indicating compositional differences between these regions [14]. Identification of the ISPs clearly demonstrates that the protein constitution of the membrane cisternae is not uniform. The ISP compartments have sharp boundaries (Figure 2B–D), suggesting that they correspond to discrete cisterna or groups thereof (Figure 10A).

ISP1 localizes to the apical cap compartment that is delimited by TgCentrin2 and thus represents the first membrane associated protein of this apical-most IMC plate. Previously, the cytoskeleton-associated proteins PhIL1 and TgDLC1 were shown to localize in part to the apical cap region [20], [22]. The C-terminal half of PhIL1 is sufficient for apical cap localization and also for retaining cytoskeletal association. This portion of the protein lacks predicted transmembrane domains or acylation signals, indicating that it links directly to a sub-domain of the cytoskeleton independent of the membrane stacks. Electron micrographs of detergent-extracted parasites show substantial differences in the cytoskeletal filaments in this region (e.g. thicker filaments and a parallel instead of interwoven arrangement), indicating that distinct sub-domains exist in both the IMC membranes and underlying network [13].

Localization of ISP2 and 3 revealed two additional sub-compartments of the IMC that have not been previously observed: a central compartment labeled by ISP2 and a basal compartment labeled by ISP3. The abutment of ISP2 and ISP3 staining against the posterior end of the apical cap likely corresponds to the junction between the apical cap and the rectangular plates constituting the remainder of the IMC. The presence of TgCentrin2 annuli at this border is striking as centrins are calcium-binding contractile proteins known to play a role in the duplication of microtubule organizing centers [39]. While the ISP3 sub-compartment clearly terminates at the posterior end of the IMC, it is unclear what accounts for the basal boundary of the ISP2 sub-compartment which lies approximately two-thirds down the length of the parasite. One possibility is an association with the cortical microtubules that also terminate in this region [40]. However, the microtubules and ISP2 signal do not consistently terminate at the same point. Alternatively, the signal termination may correspond to another junction of IMC plates and the exclusion of ISP2 from the basal region of the IMC may reflect another point of hierarchical targeting, as we discovered for ISP1 in the apical cap.

While ISP1 and 2 are both retained in mother parasites during endodyogeny, ISP3 maternal staining dissipates as daughter parasites form. The strong ISP3 signal in early buds along with the rapid attenuation of ISP3 signal in the mother during endodyogeny provides an unhampered view of the membranes of the daughter buds (Figure 2E and Video S1). Expression of IMC proteins is tightly regulated during the cell cycle including the ISPs, which show an expression profile similar to that of IMC1 (Michael White, personal communication). Thus, the bright ISP3 staining in daughters and concomitant loss of signal in mother cells could be due to synthesis in daughters and degradation in mothers. Alternatively, since palmitoylation is a reversible lipid modification, recycling by de-palmitoylation at the parent IMC and re-palmitoylation at daughter IMCs could account for the ISP3 dynamics observed.

### ISP proteins and daughter bud initiation

ISP1 and 3 are localized to numerous ring structures in oryzalin-treated parasites, indicating that initiation of bud IMC assembly repetitively occurs under these conditions and is not dependent on microtubules. Microtubule polymerization is essential for cell division and cortical microtubule extension is thought to drive bud growth, explaining why buds in parasites lacking microtubules never elongate [36]. ISP1 and 3 are localized to distinct compartments in forming daughter cells, demonstrating that IMC sub-compartmentalization is established early during endodyogeny (Video S1). The ISP1 and 3 signals are not always perfectly overlapping in oryzalin-treated cells, suggesting that IMC membrane specialization may be established even in these early bud rings, although the rings are too small to clearly visualize distinct sub-domains. The absence of ISP2 from these rings may indicate later recruitment to daughter buds or simply be a consequence of drastic perturbation of the cell under these conditions.

Some nucleating scaffold element must provide a foundation for these early IMC membrane bud rings. The earliest signs of daughter bud formation observed by electron microscopy are a dome-shaped vesicle and associated microtubules [41]. The basal complex protein TgMORN1 is the earliest protein marker of bud generation, forming a pair of rings around the centrioles after their duplication at approximately the same time daughter conoids are assembled [42]. In oryzalin-treated parasites observed during the first few hours following drug addition, initial TgMORN1 ring formation still occurs and can be followed until cells attempt to bud, at which point the inability to polymerize new microtubules results in drastic loss of parasite morphology. After 24 hours of oryzalin treatment, TgMORN1 localizes in patches sparsely associated with peripheral sheets of IMC membrane skeleton marker IMC1 but does not label anything resembling the bud rings observed for ISP1 and 3 [20], [43]. In our study, IMC1 did not localize to ISP1-labeled bud rings in oryzalin-treated parasites, demonstrating that it is not required for bud initiation. Furthermore, TgMORN1 has been disrupted and shown to be non-essential for parasite growth [44]. Future studies with ISP1 and 3 will enable the discovery of the critical nucleating factors that mediate bud initiation.

### Acylation of conserved N-terminal residues confers ISP targeting

Protein acylation is a widely employed eukaryotic mechanism to mediate membrane association of proteins that lack a transmembrane domain. Our mutation of conserved N-terminal residues that are predicted to be myristoylated and palmitoylated indicates that these modifications are responsible for IMC membrane targeting. These mutagenesis studies also agree with our deletion analysis demonstrating that the N-terminal regions of ISP1 and 3 are sufficient for correct targeting. Together, these data suggest a kinetic trapping model for ISP localization in which ISP proteins are first co-translationally myristoylated in the cytosol enabling sampling of membranes, then recognized and palmitoylated by a unique PAT (or PAT activity) that is present in each sub-compartment, thus locking the protein into the appropriate membrane sub-compartment (Figure 10B).

For ISP1 and 3, this multiple PAT model agrees with our deletion analysis showing that N-terminal regions of the proteins are sufficient for sub-compartment localization. Recognition of each ISP protein as a substrate would be determined by the context of the sequences immediately surrounding the residues required for myristoylation and palmitoylation. Indeed, additional deletion analysis showed that the first ten residues of ISP1 mostly retain apical cap targeting (data not shown). In contrast, while the N-terminal region of ISP2 is sufficient for general IMC membrane association, deletion of the C-terminal region or its substitution in the ISP2_N_/1_C_ chimera alters sub-compartment specificity. These structural changes to ISP2 may remove important information for establishing stringent PAT specificity, permitting incorporation into other IMC sub-compartments. A similar effect was recently discovered for the palmitoylated protein Vac8 in *Saccharomyces cerevisiae*. While palmitoylation of wild-type Vac8 was only catalyzed by one of the five *S. cerevisiae* PATs tested, truncation of the Vac8 C-terminus resulted in its palmitoylation by all five PATs [45]. Alternatively, it is possible that palmitoylation of the ISP family is facilitated by a single PAT that is localized throughout all three IMC compartments and regulated by additional cofactors. Modulation of PAT activity against certain substrates by additional protein cofactors has been shown in both yeast and mammalian systems [46], [47].

The presence of a Asp-His-His-Cys-cysteine-rich domain (DHHC-CRD) is the hallmark of PAT activity and has allowed for the identification of several PATs in other systems, including 7 in *S. cerevisiae* and 23 in mammalian genomes [48]. Within the *Toxoplasma* genome, 18 DHHC-CRD containing proteins are predicted to be encoded, a relatively higher number among protists (*e.g*. the *Giardia lamblia* and *Trypanosoma brucei* genomes are predicted to contain 9 and 12 PATs, respectively [49], [50]), indicating a more extensive PAT network may be present to accommodate protein sorting within the numerous unique membrane systems in apicomplexans. Future work localizing and characterizing the putative *Toxoplasma* PATs will distinguish between the possible models for ISP sorting suggested by our data.

### Hierarchical targeting of the ISP family within the IMC

Relocalization of the other ISP family members into the apical cap may explain the lack of any gross phenotype in *Δisp1* parasites. Whereas targeting to the apical cap is mediated by the N-terminal region of ISP1, relocalization of other family members into this sub-compartment is dependent on the C-terminal portion of this protein. Both the ISP1_N_/2_C_ and ISP2_N_/1_C_ chimeras support the conclusion that this gate-keeping is specific to ISP1 and directed against ISP2/3. Interestingly, while distal sequences of ISP2 are also required for its exclusion (as shown by ISP2_1–41_-HA), this is not the case for a comparable truncation of ISP3.

Perhaps the simplest explanation for the mechanism of ISP2/3 exclusion from the apical cap is provided by our multiple PAT model (Figure 10B). This model would suggest that in wild-type parasites, the presence of ISP1, either directly or indirectly via other proteins, modulates PAT activity in the apical cap, thus preventing recognition of ISP2 and 3. In the absence of the ISP1 C-terminal domain, ISP2 and 3 are able to be recognized as substrates of the apical cap PAT and also localize to this compartment. This model would also suggest that the exclusion insensitivity of truncated ISP2 (Figure 5F), as compared to truncated ISP3 (Figure 5D), may simply result from a change in the ability of PATs to specifically recognize and act upon this altered molecule (discussed in the previous section). Alternatively, deletion of ISP1 may result in relocalization of a central sub-compartment PAT into the apical cap, thus enabling ISP2 and 3 to localize to this membrane region.

Finally, it is also possible that ISP1 exclusion is the result of a receptor in the apical cap, which the C-terminal domain of ISP1 binds with a higher affinity than ISP2 or 3. The absence of the ISP1 C-terminal domain would then allow binding of the similar regions of ISP2 and 3 to the receptor in the apical cap. However, the variable exclusion observed in C-terminal truncations of ISP2 and 3 argues against this scenario. We have attempted to identify ISP1 binding partners by immunoprecipitation under gentle conditions but have had no success, indicating that if partners do exist, they are not strongly interacting. Regardless of the precise mechanism, the targeting of the ISP family demonstrates that organization of the *Toxoplasma* IMC is an interactive, complex process. To our knowledge, this hierarchical targeting is a completely unprecedented mechanism for sorting of palmitoylated proteins in any membrane system. It will be interesting to see if similar mechanisms of membrane organization are present in other members of the eukarya.

### ISP2 is important in daughter cell formation

Disruption of *ISP2* results in defects in daughter cell formation, indicating that ISP2 is important for proper coordination of daughter parasite assembly. Our observation that ∼5% of wild-type parental strain vacuoles assemble >2 daughters is in agreement with previous studies [51]. *Toxoplasma* populations have been reported to undergo flux in the percentage of parasites displaying this trait due to certain stresses [52], however the dramatic (∼60%) effects on daughter parasite assembly in the Δ*isp2* strain vastly exceed these previous reports. Furthermore, the severe fitness loss in these parasites indicates this failure to properly coordinate cell division has serious consequences for parasite biology. This could be due to abortive replication events, as we do observe ultrastructural and organelle partitioning defects that are likely terminal (e.g. parasites lacking a nucleus or apicoplast and immature daughter buds within the vacuole, Figure 9G–H). However, many of the Δ*isp2* progeny produced in parasites assembling >2 siblings appear viable as they seem to properly assembly the IMC and cortical cytoskeleton and also receive nuclear DNA, an apicoplast and a mitochondrion (data not shown). In these cases, poor control over the number of daughter cells being assembled may also render a fitness cost on parasites during the normally efficient proliferative tachyzoite life stage.

The increase in the number of daughter parasites per mother cell results in several outcomes. In some parasites, DNA replication and karyokinesis occur prior to bud formation (Figure 9E–F), while in others, multiple rounds of DNA replication appear to occur without karyokinesis, resulting in large nuclei that are segregated in a single step among multiple daughters (Figure 9D). In either case, mother parasites that produce greater than 2 daughters are no longer performing endodyogeny, but instead replicating by one form or another of endopolygeny [19], [51], [53]. The presence of replication abnormalities in Δ*isp2* parasites reminiscent of division in other *Toxoplasma* life stages and other apicomplexan species suggests this protein plays a role in coordinating progress along the proper cell division pathway in tachyzoites and that this coordination is needed to maintain parasite fitness.

It is unclear how Δ*isp2* parasites ultimately recover from these defects and return to normal growth and replication. In both of the independent *ISP2* knockouts performed months apart, the defects in growth and daughter formation were stable for at least two months. Recovery may be due to compensation via the other ISP proteins or may instead involve other players. It will be interesting to determine whether double knockouts of the ISP proteins, or even a triple knockout, will yield a more severe and stable phenotype. These functional implications for ISP2 underscore the idea that apicomplexan-specific processes are likely tied to the many hypothetical genes encoded within these parasites, some of which will provide novel therapeutic targets. The conservation of this family throughout the phylum suggests that the unique ISP targeting mechanism is conserved and raises the possibility that these proteins are more broadly involved in coordinating the various pathways of cell division that are critically important to the pathogenesis of apicomplexan parasites.

## Materials and Methods

### Ethics statement

Antibodies were raised in mice under the guidelines of the Animal Welfare Act and the PHS Policy on Humane Care and Use of Laboratory Animals. Specific details of our protocol were approved by the UCLA Animal Research Committee.

### *Toxoplasma* and host cell culture

*T. gondii* RHΔ*hpt* (parental) strain and modified strains were maintained in confluent monolayers of human foreskin fibroblast (HFF) host cells as previously described [54].

### Generation of monoclonal antibody 7E8

Monoclonal antibodies (mAb) were generated against a mixed fraction of organelles from *T. gondii* [28]. For immunization, ∼100 µg of purified organelles [55] were injected in RIBI adjuvant into a BALB/c mouse. Following four injections, the spleen was isolated, hybridoma lines were prepared, and supernatants from individual clones screened for antibody reactivity.

### Antibodies

The following primary antibodies were used in IFA or Western blot: rabbit polyclonal anti-tubulin [35], rabbit polyclonal anti-SAG1 [56], anti-IMC1 mAb 45.15 [33], anti-ROP1 mAb TG49 [57], and anti-ATrx1 mAb 11G8 [28]. Hemagglutinin (HA) epitope was detected with mAb HA.11 (Covance) or rabbit polyclonal anti-HA (Invitrogen).

### Light microscopy and image processing

Fixation and immunofluorescence staining of *T. gondii* were carried out as previously described [55]. All cells imaged in this study were formaldehyde-fixed except parasites in Figure S2B, which were fixed with methanol. Image stacks were collected at z-increments of 0.2 µm with an AxioCam MRm CCD camera and AxioVision software on an Axio Imager.Z1 microscope (Zeiss) using a 100x oil immersion objective. Deconvolved images were generated using manufacturer specified point-spread functions and displayed as maximum intensity projections.

### Identification of ISP1 by immunoaffinity purification with mAb 7E8

The protein recognized by monoclonal antibody 7E8 was isolated from 5×10^9^ *T. gondii* RH tachyzoites lysed in radioimmunoprecipitation assay (RIPA) buffer (50 mM Tris [pH 7.5], 150 mM NaCl, 0.1% sodium dodecyl sulfate [SDS], 0.5% NP-40, 0.5% sodium deoxycholate). Insoluble material was removed from the lysate by centrifugation at 10,000× *g* for 30 min after which the remaining soluble lysate fraction was incubated with mAb 7E8 cross-linked to protein G-Sepharose beads (Amersham) using dimethylpimelimidate as previously described [58]. After washing in RIPA buffer, the bound protein was eluted using high pH (100 mM triethylamine, pH 11.5) and the eluate was separated by SDS-polyacrylamide gel electrophoresis (PAGE). Coomassie staining identified a single 18-kDa band, which was excised and trypsin digested before analysis by mass spectrometry at the Vincent Coates Foundation Mass Spectrometry Laboratory, Stanford University Mass Spectrometry (http://mass-spec.stanford.edu).

### Expression of epitope-tagged and fluorescent fusion proteins

YFP-αTubulin and mRFP-TgCentrin2 were expressed in parasites using previously described plasmids [20], [59]. HA epitope-tagged lines and YFP fusions pISP1/2/3-HA/YFP were generated by cloning the genomic loci of ISP1 (primers P1/P2), ISP2 (primers P3/P4) or ISP3 (primers P5/P6) into the expression plasmids pNotI-HA-HPT or pNotI-YFP-HPT using the restriction sites *HindIII/NotI*. These vectors contain a C-terminal HA tag or YFP fusion and selectable marker *HPT* driven by the *DHFR* promoter [60]. The ISP1_1–65_ truncation was generated by cloning YFP (primers P7/P8) at the restriction sites *EcoRV/PacI* in pISP1-YFP. The ISP2_1–41_ truncation was generated by cloning YFP (primers P9/P8) at the restriction sites *RsrII/NotI* in pISP2-YFP. The ISP3_1–36_ truncation was generated by cloning the ISP3 promoter and residues 1–36 (primers P10/P11) at the restriction sites *PmeI/AvrII* in the previously described vector ptubYFP-YFP/sagCAT [61]. The ISP1_64–176_ truncation was generated by cloning the ISP1 promoter and start codon (primers P1/P12) at the restriction sites *HindIII/EcoRV* in pISP1-YFP. The ISP1_N_/2_C_ chimera was generated by cloning ISP2_43–160_ (primers P13/P4) at the restriction sites *EcoRV/NotI* in pISP1-YFP. The ISP2_N_/1_C_ chimera was generated by cloning ISP1_67–176_-YFP (primers P14/P8) at the restriction sites *RsrII/PacI* in pISP2-HA. For expression, 1.6×10^7^ parasites were transfected with 30 µg of plasmid and then analyzed by IFA as specified in figure legends.

### Alpha-toxin treatment

Separation of the parasite IMC and plasma membrane was achieved by treatment with *C. septicum* alpha-toxin as previously described [33]. Briefly, freshly lysed, extracellular parasites were washed and incubated 4 hrs in serum free media with or without 20 nM activated alpha-toxin. Following treatment, cells were fixed in 3.5% formaldehyde, allowed to settle on glass slides and analyzed by IFA.

### Disruption of the cortical cytoskeleton of *T. gondii*

Tachyzoites were allowed to infect HFF monolayers on coverslips in media containing 0.5 or 2.5 µM oryzalin (Sigma). Parasites were allowed to grow 30–40 hrs post-infection and then fixed and examined by IFA.

### Generation of ISP2 and ISP3 antisera

The coding sequences for ISP2 (primers P15/P16) and ISP3 (primers P17/P18) were PCR amplified from *T. gondii* cDNA and cloned into pET101/D-TOPO (Invitrogen). Constructs were transformed into *E. coli* BL21DE3 cells, grown to A_600_ of 0.6–0.8 and induced with 1 mM isopropyl 1-thio-β-D-galactopyranoside (Sigma) for 5 hrs at 37°C. Recombinant ISP2 and ISP3 were purified over Qiagen Ni-NTA agarose under denaturing conditions and eluted with a low-pH buffer as per the manufacturer's instructions. Eluted proteins were dialyzed against PBS and ∼75 µg was injected per immunization into BALB/c mice (Charles River) on a 21 day immunization schedule. Polyclonal antiserum was collected from mice after the second boost and screened by IFA and Western blot analysis.

### Detergent extraction of ISP proteins

For detergent extraction experiments, 3×10^7^ freshly lysed parasites were washed in PBS, pelleted and lysed in 1 mL TBS (50 mM Tris-HCl [pH 7.4], 150 mM NaCl) containing 0.5% NP-40 and complete protease inhibitors (Roche) for 15 min at 4°C and then centrifuged for 15 min at 14,000× *g*. Equivalent amounts of total, supernatant and pellet fractions were separated on a 15% gel, transferred to nitrocellulose and blotted using anti-IMC1, anti-ROP1, mAb 7E8, polyclonal anti-ISP2, and polyclonal anti-ISP3.

### Site directed mutagenesis

Mutations were generated by Quick Change Mutagenesis (Strategene) using HA-tagged, wild-type ISP1, 2 or 3 with mutagenesis primers as follows (forward primer given, reverse compliment was also used): ISP1: G2A (P19), C7S (P20), C8S (P21), C7,8S (P22). ISP2: G2A (P23), C5S (P24), C8S (P25), C9S (P26), C8,9S (P27), C5,8,9S (P28). ISP3: G2A (P29), C6S (P30), C7S (P31), C6,7S (P32). PCR amplified products were treated with *DpnI* to digest wild-type template and transformed into *E. coli*. Recovered clones were sequenced to confirm mutations.

### Disruption of *ISP1*

The deletion of the *ISP1* gene was accomplished by double homologous recombination using a construct derived from the pMini-GFP.ht knockout vector [62] which contains the selectable marker hypoxanthine-xanthine-guanine phosphoribosyltransferase (*HPT*) and also contains the green fluorescent protein (*GFP*) as a downstream marker to distinguish homologous and heterologous recombinants. The 5′ flank (3,147 bp) and 3′ flank (3,042 bp) of *ISP1* were amplified from strain RH genomic DNA using primer pairs P33/P34 and P35/P36, respectively. These genomic flanks were then cloned into pMini-GFP.ht upstream and downstream of *HPT*, resulting in the vector pISP1-KO-HPT.

After linearization with *NheI*, 30 µg of pISP1-KO-HPT was transfected into RHΔ*hpt* parasites and selection for *HPT* was applied 12 hours post-transfection using 50 µg/ml mycophenolic acid and 50 µg/ml xanthine. Surviving parasites were cloned by limiting dilution eight days post-transfection and screened for GFP by fluorescence microscopy. GFP-negative clones were assessed for absence of mAb 7E8 staining by IFA. Western blot analysis was carried out on whole-cell lysates of Δ*isp1* clones and parental strains using mAb 7E8 and anti-ROP1 antibody as previously described [55]. The *HPT* gene was removed from RHΔ*isp1* + *HPT* by a second round of double homologous recombination. The pISP1-KO-HPT vector was digested by *EcoRV/NheI* to remove the *HPT* gene and then blunted using Klenow enzyme and re-circularized by ligation. The resulting vector was linearized by *EcoRI* and transformed into RHΔ*isp1* + *HPT*, followed by selection for the absence of *HPT* on 200 µg/ml 6-thioxanthine (Sigma). After 3 weeks of selection, parasites were cloned and screened for the absence of GFP expression. Clones that were GFP-negative were then assessed for the inability to grow in mycophenolic acid and xanthine, indicating loss of *HPT*. One such clone was chosen and deletion of the *ISP1* locus was confirmed by PCR. This clone was designated Δ*isp1*.

### Generation of Δ*ku80*Δ*hpt* strain parasites

The *HPT* selectable marker was removed from the *Ku80* locus of the previously described Δ*ku*80 strain [38]. Briefly, 10 µg of a PCR fusion construct containing a 5′ *Ku80* flank (primers P37/P38) fused to a 3′ *Ku80* flank (primers P39/P40) was transfected into RHΔ*ku*80-*HPT* parasites. Selection against *HPT* with 6-thioxanthine and confirmation of marker loss were carried out as described above.

### Disruption of *ISP2* and *ISP3*

For disruption of *ISP*2, a knockout vector was generated by cloning ∼3 kb 5′ (primers P41/P42) and 3′ (primers P43/P44) genomic flanks into a modified version of pMiniGFP.ht in which *HPT* was replaced by the selectable marker *DHFR-TSc3*, yielding the vector pISP2KO-DHFR-TSc3. After linearization by *NotI*, 30 µg of this vector was transfected into Δ*ku80*Δ*hpt* parasites and selection was applied 12 hours post-transfection using 1 µM pyrimethamine. Parasites were cloned and confirmed to lack ISP2 as described above. For disruption of ISP3, the vector pISP3-KO-HPT was generated by cloning ∼3 kb 5′ (primers P45/P46) and 3′ (primers P47/P48) genomic flanks into pMiniGFP.ht. After linearization by *KpnI* and transfection into the Δ*ku80*Δ*hpt* strain, parasites were selected for *HPT*, cloned and confirmed to lack ISP3 as described above.

### Competition growth analysis of Δ*isp2* parasites

Freshly lysed parental and Δ*isp2* parasites were counted and mixed in desired ratios before infection of 3.3×10^6^ parasites into a T25 flask of confluent HFFs. Parasites were allowed to disrupt the monolayer before passing into a fresh T25. At initial infection and at each passage, samples of the mixed culture were infected into coverslips and allowed to grow 32 hours before fixation and staining with polyclonal anti-ISP2 and rabbit polyclonal anti-tubulin as a co-marker to monitor mixed culture composition. At least 500 vacuoles were counted from each of 4 coverslips per passage. Values represent mean 3 standard deviations for a representative experiment.

### Quantification of aberrant numbers of daughter parasite assembly

Parental line and Δ*isp2* parasites were infected onto coverslips and allowed to grow 18–24 hours before fixation and staining with mAb 7E8 as a marker for daughter buds and rabbit polyclonal anti-tubulin as a co-marker. Fifty vacuoles containing parasites undergoing bud formation were counted from each of 3 coverslips per sample. Vacuoles containing one or more parasites assembling >2 daughters were scored as aberrant. Values represent the mean ± SD from a representative experiment.

## Supporting Information

Figure S1 ISP ortholog groups. OrthoMCL DB (www.orthomcl.org) was utilized to identify ortholog groups for the ISP family. ISP1 and 2 belong to one OrthoMCL group (OG4_23348) (A) while ISP3 belongs to another group (OG4_34375) (B). The ISP1 and 2 group contains proteins from all apicomplexans available in the OrthoMCL DB while the ISP3 group contains only proteins from *Neospora caninum, Plasmodium* species, and *Babesia bovis*. EuPathDb (www.eupathdb.org) accession numbers are given for each protein. The *P. berghei* protein PB301233.00.0 and *P. yoelii* protein PYO2085 each lack a start methionine, indicating incomplete N-termini in the annotation of the gene models associated with these proteins. The related CP15/60 protein from *Cryptosporidia* forms a separate OrthoMCL group (OG4_74892, data not shown).
(9.69 MB TIF)

Figure S2 Antibody confirmation of sub-compartment localizations for endogenous ISP2 and ISP3. **A.** ISP2 antisera confirms the localization of endogenous ISP2 to the central IMC sub-compartment in a fashion identical to the HA epitope-tagged ISP2 shown in Figure 2B. Endogenous ISP2 is clearly absent from the apical cap (brackets) and basal portion of the IMC. Red: polyclonal anti-ISP2 detected by Alexa594-anti-mouse IgG. Green: anti-tubulin antibody detected by Alexa488-anti-rabbit IgG. **B-C.** ISP3 antisera functions poorly by IFA. The staining is, however, sufficient to (B) confirm the localization of endogenous ISP3 to the central and basal IMC shown by HA epitope-tagged ISP3 in Figure 2C and (C) confirm the attenuation of maternal ISP3 signal and enrichment of ISP3 in daughter parasites during endodyogeny as shown in Figure 2E. Red: polyclonal anti-ISP3 detected by Alexa594-anti-mouse IgG. Green: anti-tubulin antibody detected by Alexa488-anti-rabbit IgG.
(4.24 MB TIF)

Figure S3 Mutation of ISP2 residues predicted for acylation results in ISP2 mistargeting. Mutations of residues predicted for myristoylation or palmitoylation were generated in an HA epitope-tagged copy of ISP2 and expressed in parasites under the control of the endogenous promoter. A severe targeting defect occurs in ISP2 (G2A) in which ISP2 signal is dispersed throughout the cell in a punctate fashion. The ISP2 (C5S) mutant shows an intermediate localization defect with some mistargeting and some proper localization. Mutation of the conserved cysteine pair residues individually or together does not grossly mistarget ISP2 (C8S), (C9S), or (C8,9S). A serious targeting defect occurs when all three N-terminal cysteines are coordinately mutated in ISP2 (C5,8,9S). While ISP2 (C5,8,9S) is distributed throughout the cytosol, signal concentration is observed perinuclear and just apical of the nucleus (arrows). Red: anti-HA antibody detected by Alexa594-anti-mouse IgG. Green: anti-tubulin antibody detected by Alexa488-anti-rabbit IgG. Blue: Hoechst stain.
(7.83 MB TIF)

Figure S4 Mutation of ISP3 residues predicted for acylation results in ISP3 mistargeting. Mutations of residues predicted for myristoylation or palmitoylation were generated in an HA epitope-tagged copy of ISP3 and expressed in parasites under the control of the endogenous promoter. A severe targeting defect occurs in ISP3 (G2A) with the mutant protein dispersed throughout the cell in a punctate fashion. Individual cysteine mutants ISP3 (C6S) and (C7S) show no gross defect in targeting. Coordinated mutation of these cysteines results in gross mistargeting of ISP3 (C6,7S) throughout the cell in a punctate fashion. As seen in ISP1 and ISP2 coordinated cysteine mutants, a concentration of signal is observed just apical of the nucleus (arrows). Red: anti-HA antibody detected by Alexa594-anti-mouse IgG. Green: anti-tubulin antibody detected by Alexa488-anti-rabbit IgG. Blue: Hoechst stain.
(6.36 MB TIF)

Figure S5 Disruption of *ISP3*. Western blot analysis using polyclonal anti-ISP3 confirms the loss of ISP3 in *Δisp3* parasites. ROP1 serves as a loading control.
(0.49 MB TIF)

Table S1 Primers used in this study as discussed in text. Restriction sites and mutated bases are shown in lowercase.
(2.29 MB TIF)

Video S1 ISP1 and 3 during *Toxoplasma* endodyogeny. Parasites stably expressing ISP3-HA were allowed to infect HFFs and grow 24 hrs before fixation and IFA analysis. Serial sections were acquired, deconvolved and projected as a three-dimensional image. Visualization of ISP1 and ISP3 during endodyogeny shows that ISP1 is present in the maternal IMC apical cap as well as in the apical cap of each forming daughter. ISP3 exhibits different dynamics during endodyogeny: while it is clearly seen in the central and basal IMC compartments of each forming daughter, the signal has disappeared from the maternal IMC. At this stage of endodyogeny, the replicated nucleus is being segregated between the two daughter buds forming within each mother. The distinct compartments of the IMC revealed by ISP1 and 3 are already clearly visible. Red: mAb 7E8 detected by Alexa594-anti-mouse IgG. Green: anti-HA antibody detected by Alexa488-anti-rabbit IgG. Blue: Hoechst stain.
(2.02 MB MOV)

Video S2 ISP1 early bud rings in oryzalin treated *Toxoplasma*. Parasites stably expressing ISP1-HA were allowed to infect HFFs in the presence of 0.5 µM oryzalin and grow 30 hours before fixation and IFA analysis. Serial sections were acquired, deconvolved and projected as a three-dimensional image. In the absence of cortical microtubules, a single mother parasite repeatedly fails to undergo productive replication. Numerous ISP1-labeled rings (red) are found clustered in the center of the cell. IMC1, shown in green, does not co-localize with these structures. Red: anti-HA antibody detected by Alexa594-anti-rabbit IgG. Green: anti-IMC1 antibody detected by Alexa488-anti-mouse IgG.
(1.57 MB MOV)
