# Supplementary figures and images for: A Novel Family of Toxoplasma IMC Proteins Displays a Hierarchical Organization and Functions in Coordinating Parasite Division
Source: PLoS Pathog. 2010 Sep 9;6(9):e1001094. doi: 10.1371/journal.ppat.1001094 (PMC2936552; doi:10.1371/journal.ppat.1001094)

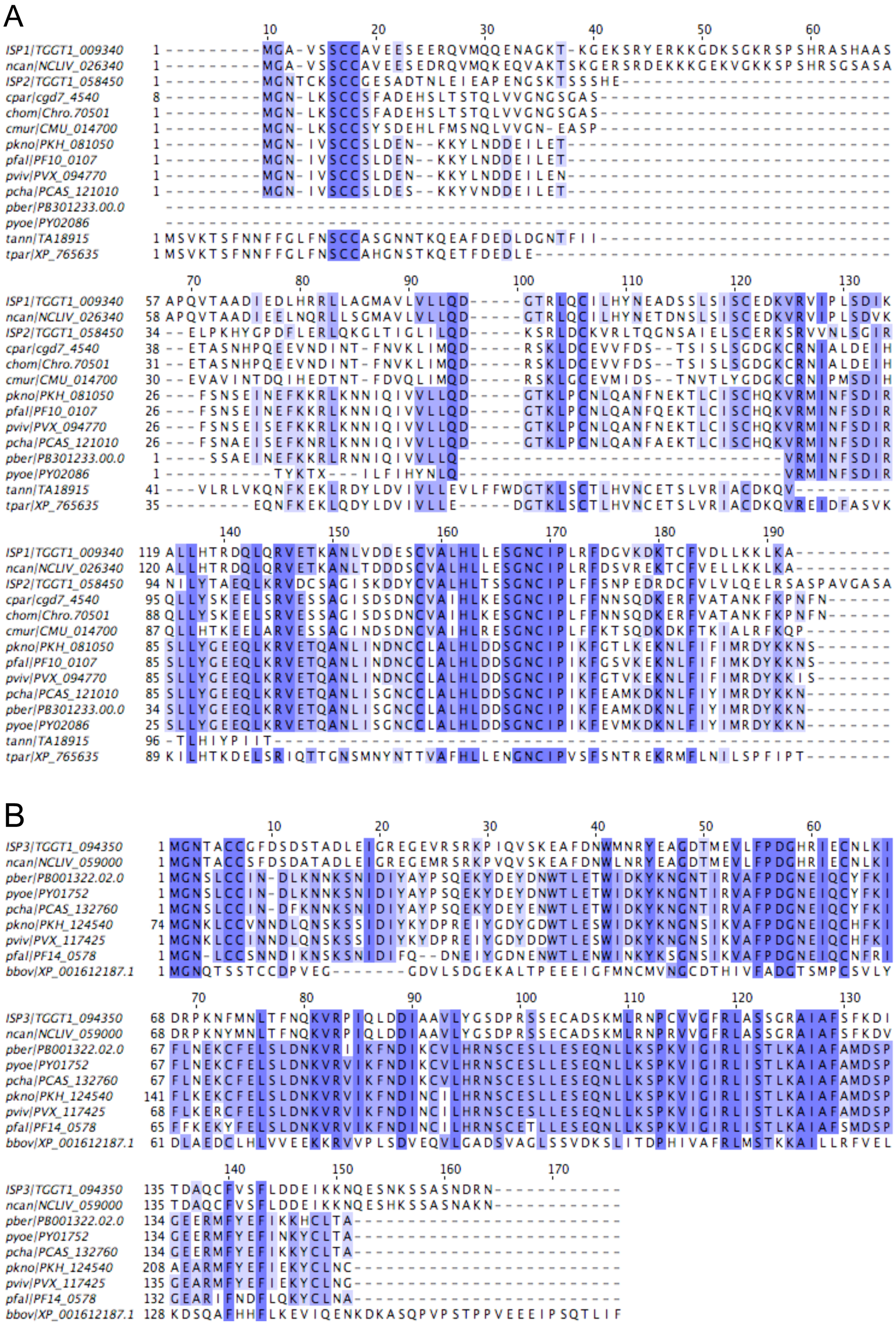

Supplement: Figure S1 — ISP ortholog groups. OrthoMCL DB (www.orthomcl.org) was utilized to identify ortholog groups for the ISP family. ISP1 and 2 belong to one OrthoMCL group (OG4_23348) (A) while ISP3 belongs to another group (OG4_34375) (B). The ISP1 and 2 group contains proteins from all apicomplexans available in the OrthoMCL DB while the ISP3 group contains only proteins from Neospora caninum, Plasmodium species, and Babesia bovis. EuPathDb (www.eupathdb.org) accession numbers are given for each protein. The P. berghei protein PB301233.00.0 and P. yoelii protein PYO2085 each lack a start methionine, indicating incomplete N-termini in the annotation of the gene models associated with these proteins. The related CP15/60 protein from Cryptosporidia forms a separate OrthoMCL group (OG4_74892, data not shown). (9.69 MB TIF) [file ppat.1001094.s001.tif]

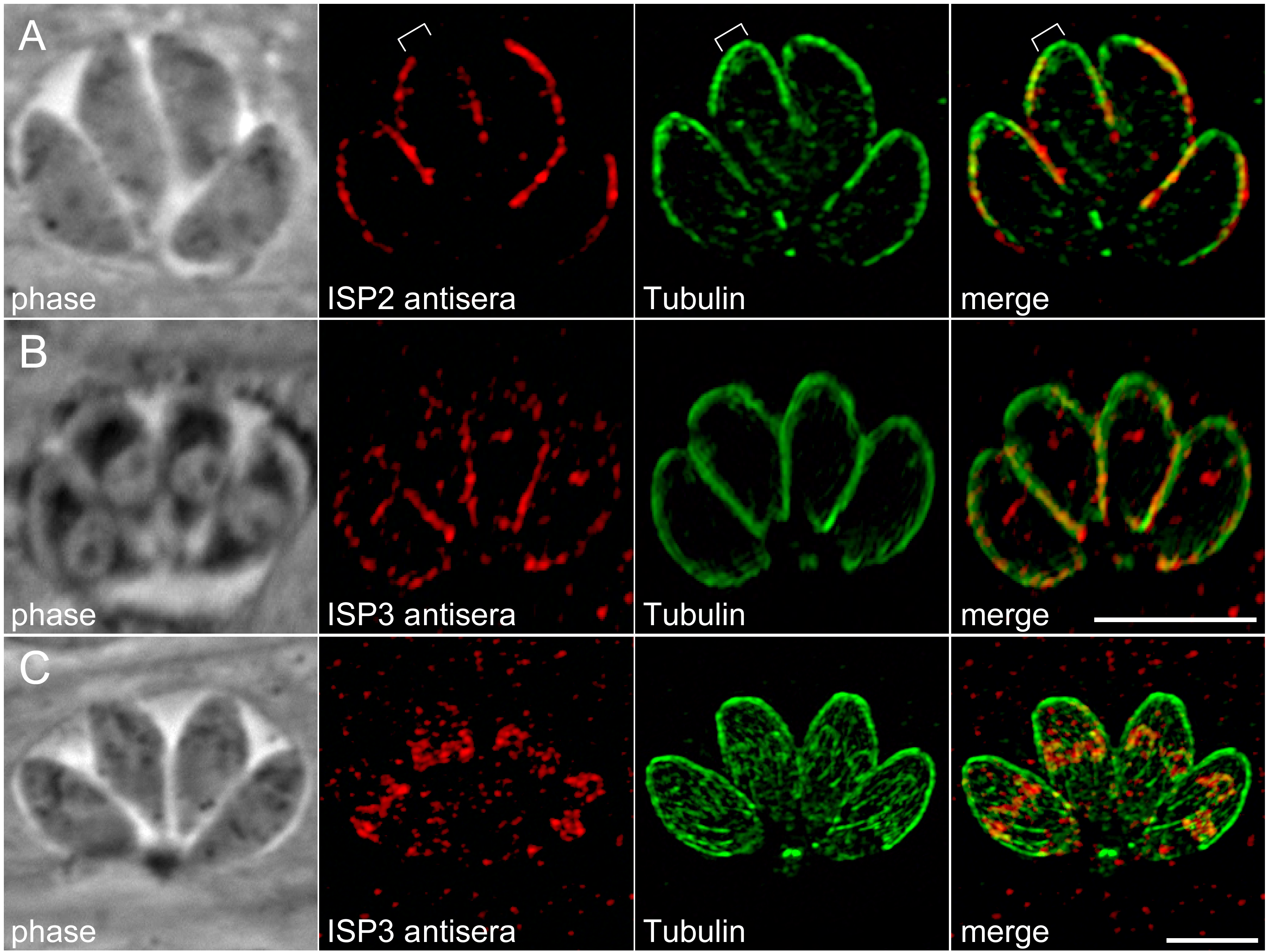

Supplement: Figure S2 — Antibody confirmation of sub-compartment localizations for endogenous ISP2 and ISP3. A. ISP2 antisera confirms the localization of endogenous ISP2 to the central IMC sub-compartment in a fashion identical to the HA epitope-tagged ISP2 shown in Figure 2B. Endogenous ISP2 is clearly absent from the apical cap (brackets) and basal portion of the IMC. Red: polyclonal anti-ISP2 detected by Alexa594-anti-mouse IgG. Green: anti-tubulin antibody detected by Alexa488-anti-rabbit IgG. B-C. ISP3 antisera functions poorly by IFA. The staining is, however, sufficient to (B) confirm the localization of endogenous ISP3 to the central and basal IMC shown by HA epitope-tagged ISP3 in Figure 2C and (C) confirm the attenuation of maternal ISP3 signal and enrichment of ISP3 in daughter parasites during endodyogeny as shown in Figure 2E. Red: polyclonal anti-ISP3 detected by Alexa594-anti-mouse IgG. Green: anti-tubulin antibody detected by Alexa488-anti-rabbit IgG. (4.24 MB TIF) [file ppat.1001094.s002.tif]

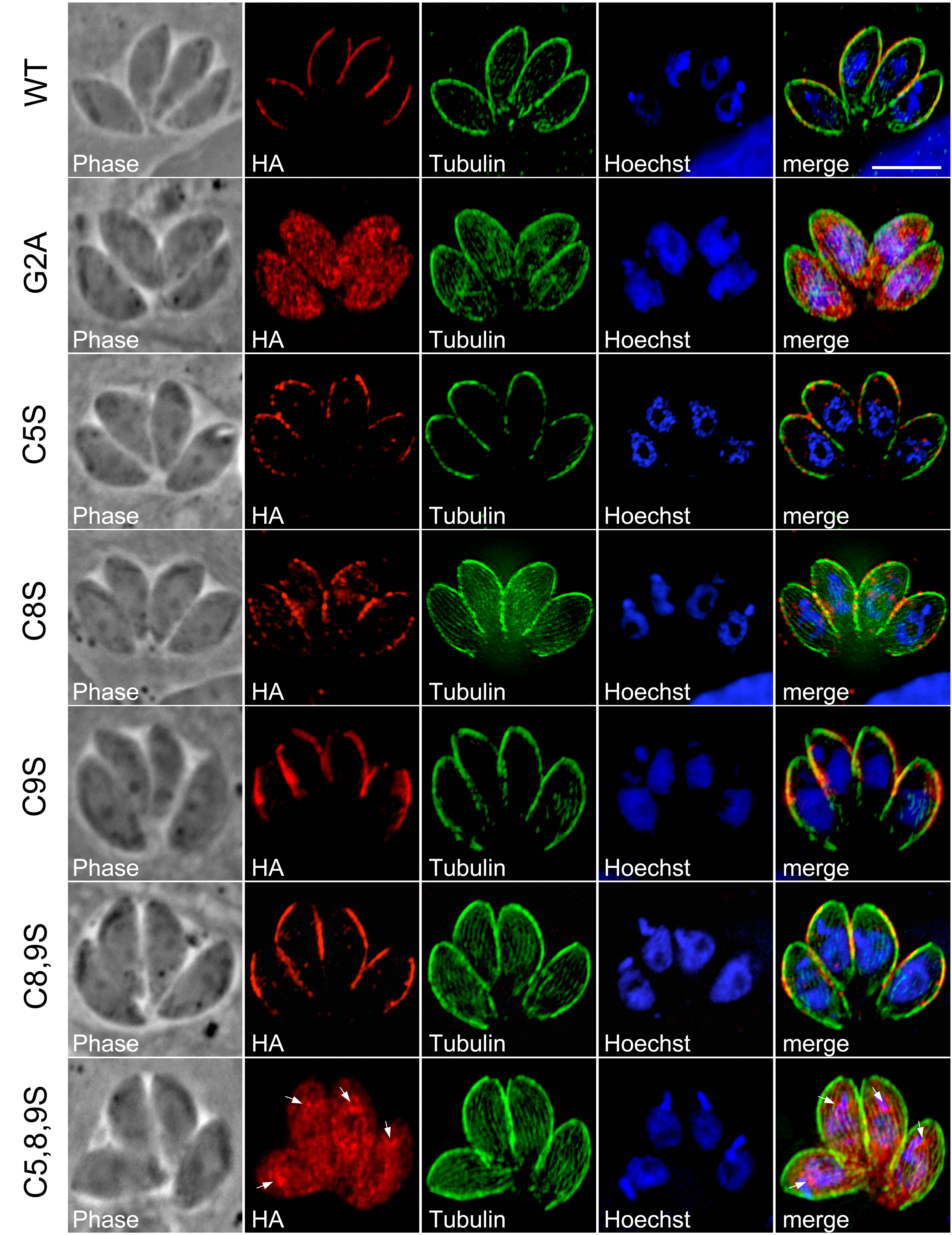

Supplement: Figure S3 — Mutation of ISP2 residues predicted for acylation results in ISP2 mistargeting. Mutations of residues predicted for myristoylation or palmitoylation were generated in an HA epitope-tagged copy of ISP2 and expressed in parasites under the control of the endogenous promoter. A severe targeting defect occurs in ISP2 (G2A) in which ISP2 signal is dispersed throughout the cell in a punctate fashion. The ISP2 (C5S) mutant shows an intermediate localization defect with some mistargeting and some proper localization. Mutation of the conserved cysteine pair residues individually or together does not grossly mistarget ISP2 (C8S), (C9S), or (C8,9S). A serious targeting defect occurs when all three N-terminal cysteines are coordinately mutated in ISP2 (C5,8,9S). While ISP2 (C5,8,9S) is distributed throughout the cytosol, signal concentration is observed perinuclear and just apical of the nucleus (arrows). Red: anti-HA antibody detected by Alexa594-anti-mouse IgG. Green: anti-tubulin antibody detected by Alexa488-anti-rabbit IgG. Blue: Hoechst stain. (7.83 MB TIF) [file ppat.1001094.s003.tif]

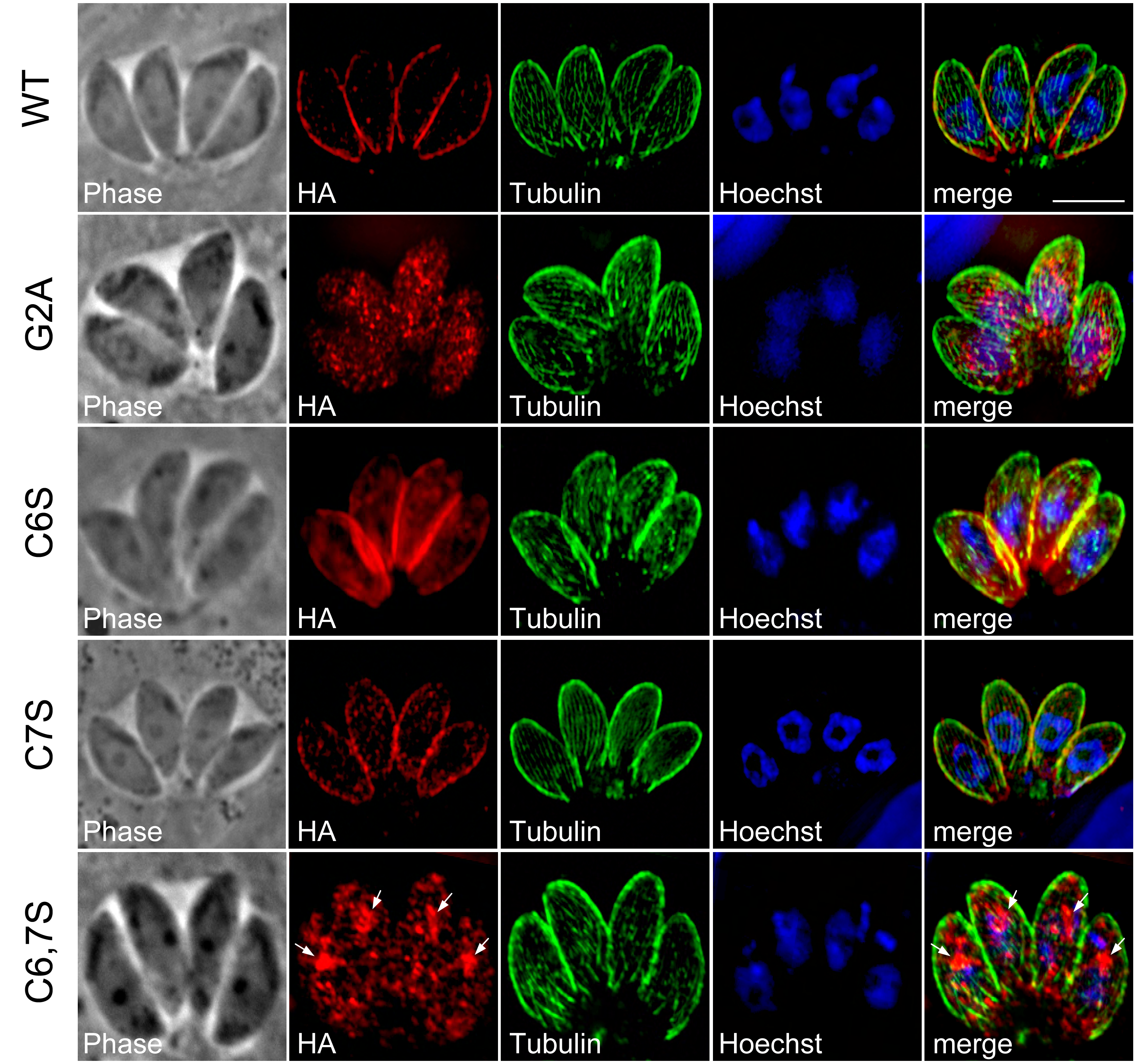

Supplement: Figure S4 — Mutation of ISP3 residues predicted for acylation results in ISP3 mistargeting. Mutations of residues predicted for myristoylation or palmitoylation were generated in an HA epitope-tagged copy of ISP3 and expressed in parasites under the control of the endogenous promoter. A severe targeting defect occurs in ISP3 (G2A) with the mutant protein dispersed throughout the cell in a punctate fashion. Individual cysteine mutants ISP3 (C6S) and (C7S) show no gross defect in targeting. Coordinated mutation of these cysteines results in gross mistargeting of ISP3 (C6,7S) throughout the cell in a punctate fashion. As seen in ISP1 and ISP2 coordinated cysteine mutants, a concentration of signal is observed just apical of the nucleus (arrows). Red: anti-HA antibody detected by Alexa594-anti-mouse IgG. Green: anti-tubulin antibody detected by Alexa488-anti-rabbit IgG. Blue: Hoechst stain. (6.36 MB TIF) [file ppat.1001094.s004.tif]

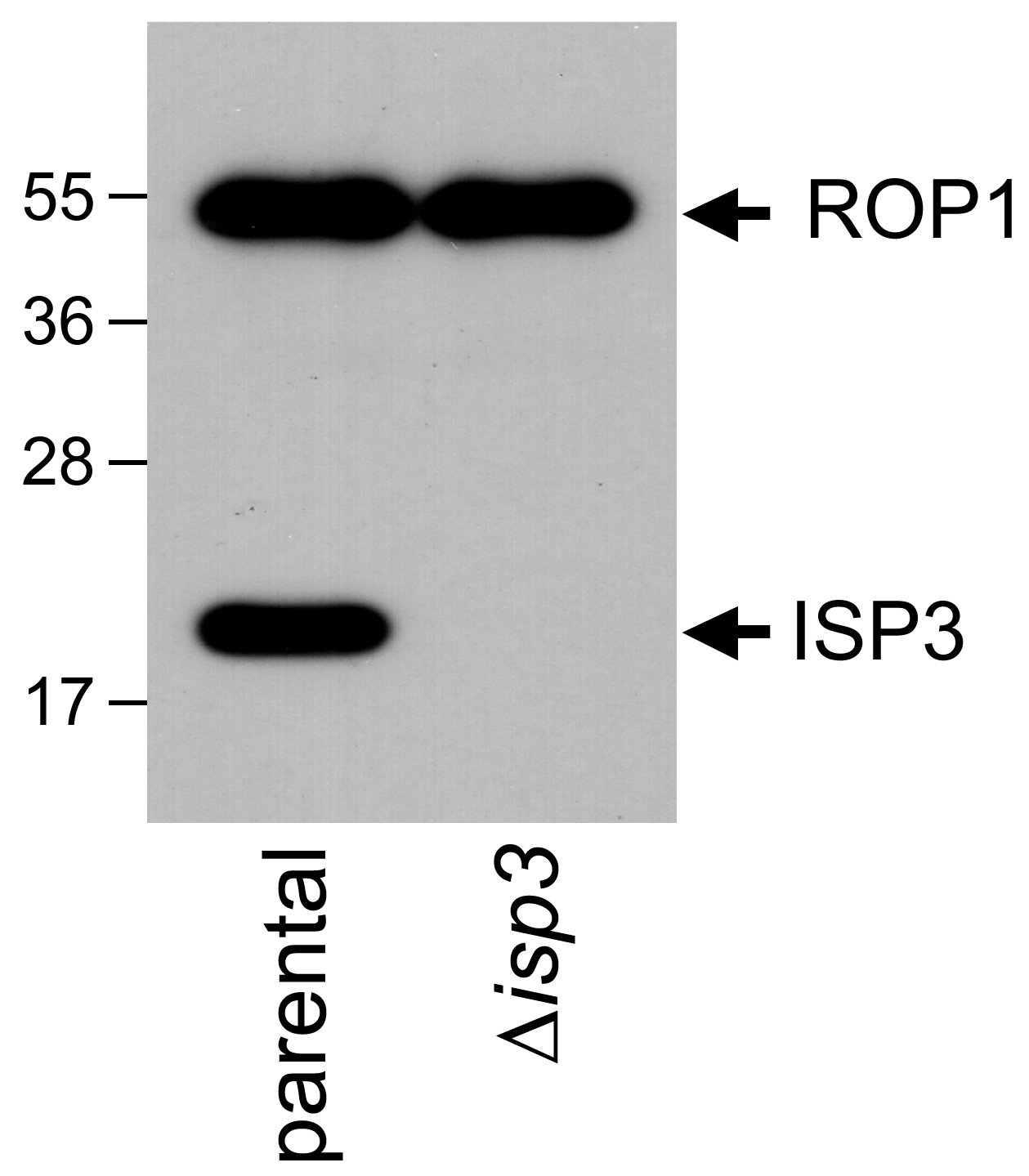

Supplement: Figure S5 — Disruption of ISP3. Western blot analysis using polyclonal anti-ISP3 confirms the loss of ISP3 in Δisp3 parasites. ROP1 serves as a loading control. (0.49 MB TIF) [file ppat.1001094.s005.tif]

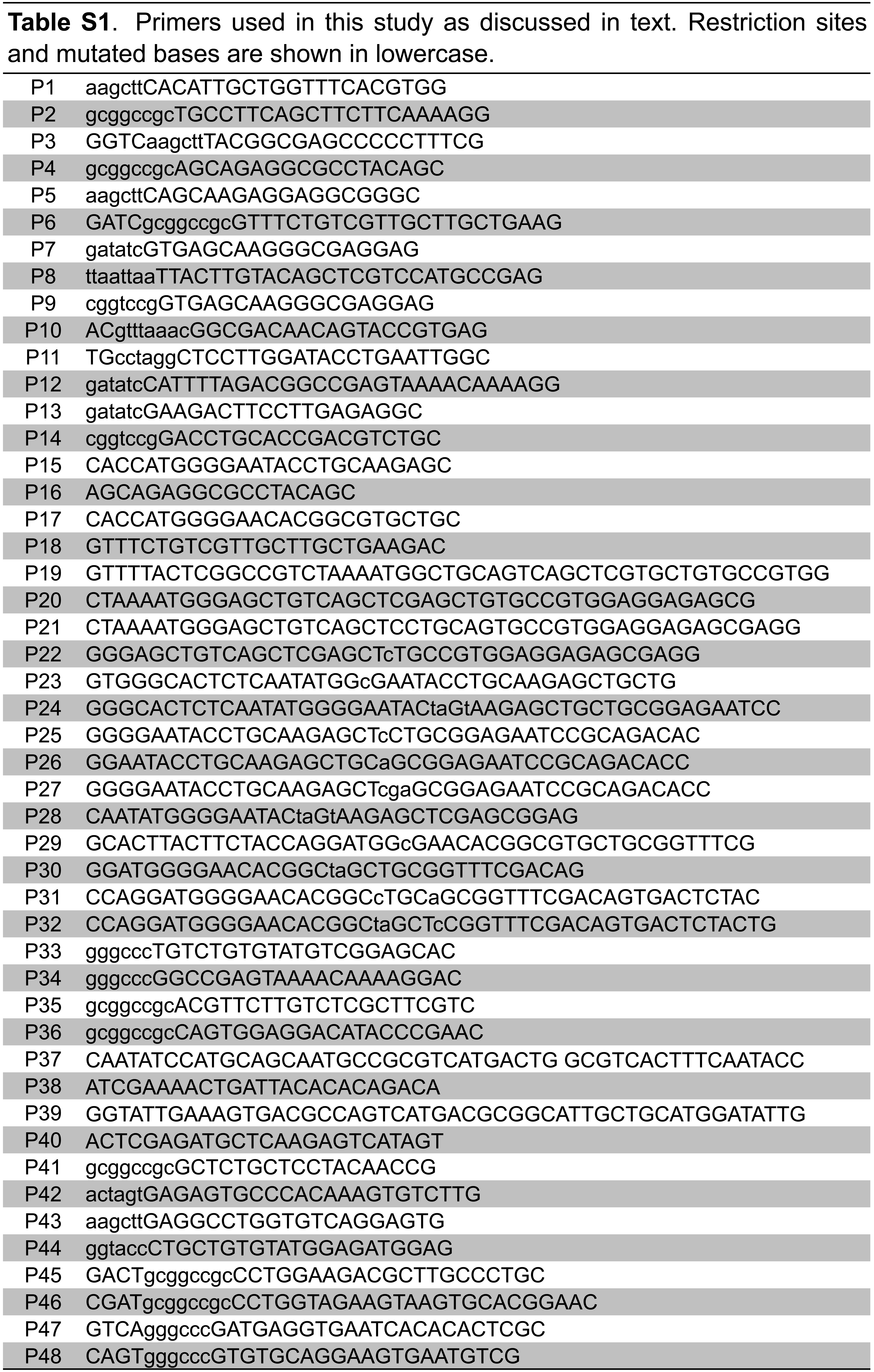

Supplement: Table S1 — Primers used in this study as discussed in text. Restriction sites and mutated bases are shown in lowercase. (2.29 MB TIF) [file ppat.1001094.s006.tif]
